# The ESKAPE mobilome contributes to the spread of antimicrobial resistance and CRISPR-mediated conflict between mobile genetic elements

**DOI:** 10.1093/nar/gkac1220

**Published:** 2023-01-05

**Authors:** João Botelho, Adrian Cazares, Hinrich Schulenburg

**Affiliations:** Antibiotic Resistance Evolution Group, Max Planck Institute for Evolutionary Biology, Plön, Germany; Department of Evolutionary Ecology and Genetics, Zoological Institute, Christian Albrechts University, Kiel, Germany; EMBL’s European Bioinformatics Institute (EMBL-EBI), Wellcome Genome Campus, Cambridge, UK; Wellcome Sanger Institute, Wellcome Genome Campus, Cambridge, UK; Antibiotic Resistance Evolution Group, Max Planck Institute for Evolutionary Biology, Plön, Germany; Department of Evolutionary Ecology and Genetics, Zoological Institute, Christian Albrechts University, Kiel, Germany

## Abstract

Mobile genetic elements (MGEs) mediate the shuffling of genes among organisms. They contribute to the spread of virulence and antibiotic resistance (AMR) genes in human pathogens, such as the particularly problematic group of ESKAPE pathogens. Here, we performed the first systematic analysis of MGEs, including plasmids, prophages, and integrative and conjugative/mobilizable elements (ICEs/IMEs), across all ESKAPE pathogens. We found that different MGE types are asymmetrically distributed across these pathogens, and that most horizontal gene transfer (HGT) events are restricted by phylum or genus. We show that the MGEs proteome is involved in diverse functional processes and distinguish widespread proteins within the ESKAPE context. Moreover, anti-CRISPRs and AMR genes are overrepresented in the ESKAPE mobilome. Our results also underscore species-specific trends shaping the number of MGEs, AMR, and virulence genes across pairs of conspecific ESKAPE genomes with and without CRISPR-Cas systems. Finally, we observed that CRISPR spacers found on prophages, ICEs/IMEs, and plasmids have different targeting biases: while plasmid and prophage CRISPRs almost exclusively target other plasmids and prophages, respectively, ICEs/IMEs CRISPRs preferentially target prophages. Overall, our study highlights the general importance of the ESKAPE mobilome in contributing to the spread of AMR and mediating conflict among MGEs.

## INTRODUCTION

Mobile genetic elements (MGEs) are DNA entities that are capable of capturing and shuffling genes intra- and intercellularly (1). Coevolution of bacterial hosts with these MGEs has driven the evolution of complexity (2). Movement within the genome is often mediated by specific MGEs, such as insertion sequences and transposons (3). Others like plasmids, prophages, and integrative and conjugative/mobilizable elements (ICEs/IMEs) are key vectors for intercellular mobility, being responsible for a large fraction of the variability observed between bacterial species (4–7). Bacteria undergo extensive horizontal gene transfer (HGT), and some estimates suggest that more than 80% of bacterial genes were horizontally transferred at some point in their evolutionary history (8). These events are largely shaped by ecological niches, by the difference in the GC content between pairs of bacteria exchanging material, and by phylogenetic barriers (9–11). Network-based methods are useful to trace HGT events and recover shared content between bacterial genomes (11,12), and have been recently applied to explore the population structure of thousands of plasmids (13,14). Even though this approach has been useful to explore population structure of plasmids, the study of potential HGT events involving other MGEs (such as prophages and ICEs/IMEs) is largely unexplored. Moreover, only a few studies have used network-based approaches to explore the co-evolutionary dynamics of different MGE types (15–17).

MGEs carry non-essential genes that can provide their bacterial host with adaptive functions and alter their fitness, such as antimicrobial resistance (AMR) and virulence genes (18,19). These elements employ a myriad of ecological and evolutionary strategies to promote their own replication and transmission, which allow them to persist even in the absence of positive selection for the beneficial genes they carry. For example, non-mobilizable plasmids can persist over evolutionary timescales without selection for the plasmid function, while multicopy plasmids can promote the coexistence of ancestral and novel functions, allowing bacteria to escape from fitness trade-offs (20,21). Bacteria have developed a variety of, often complex, defense mechanisms against invading MGEs, including restriction-modification and clustered regularly interspaced short palindromic repeats (CRISPR) and CRISPR-associated (Cas) genes (22,23). These systems are usually clustered in ‘defense islands’ and are widespread in bacteria and archaea (24,25). Partially reconstructing recent HGT events is made possible by the repeated incorporation of spacer sequences, which are derived from fragments of invading MGEs, into CRISPR loci. When dealing with obligatory parasites, CRISPR-Cas immunity that inhibits HGT can be advantageous, but it can also be harmful since it blocks the acquisition of novel genetic traits carried by MGEs. Invasion by these MGEs can still be associated with fitness costs that may lead to selection against carriage (5,26). Hence, bacteria often face a trade-off between immunity and acquisition of novel elements, which favour adaptation to different ecological niches and stressors, such as antibiotic pressure. MGEs can be equipped with inhibitors of CRISPR-Cas systems, called anti-CRISPR (Acr) proteins, which have been reported mostly in prophages (27–29). Recently, Acr proteins were identified in non-phage MGEs, including plasmids and ICEs (30).

Bacterial pathogens belonging to the ESKAPE panel consist of five species (*Enterococcus faecium*, *Staphylococcus aureus*, *Klebsiella pneumoniae*, *Acinetobacter baumannii* and *Pseudomonas aeruginosa*) and one genus (*Enterobacter* sp.) (31,32). These pathogens are frequently involved in problematic nosocomial infections, due to their multi-drug resistance and/or invasive phenotypes (33–38). The WHO recently published a list of pathogens for which new antibiotic development is urgently required, and the ESKAPE pathogens were designated ‘priority status’ (39). AMR and virulence genes are broadly distributed in plasmids across the ESKAPE pathogens (19,33), and also in ICEs (40,41). Recently, CRISPR-Cas systems have been identified in plasmids and ICEs from several bacterial species (including representatives of the ESKAPE pathogens), and may be involved in conflict between MGEs (42–44).

In this study, we performed the first systematic analysis of the ESKAPE pathogens mobilome. We asked (i) how prevalent are different MGEs (prophages, ICEs/IMEs and plasmids) across the ESKAPE pathogens; (ii) how broad or constrained is the combined MGEs’ network; (iii) which functions are overrepresented in these MGEs, and if AMR and virulence genes are differently distributed in pairs of conspecific ESKAPE pathogens with and without CRISPR-Cas systems, which we here focus on as examples of effective defense systems in bacteria (22,25); (iv) whether the CRISPR spacers have a targeting bias towards different MGE types, i.e. prophages, plasmids, and ICEs/IMEs. We found that plasmids, ICEs/IMEs, and prophages are unequally distributed across these pathogens, and found signatures of HGT between different species. Uncovering the structure of MGEs and masked (i.e. MGE-free) genomes allowed us to discover an overrepresentation of AMR genes and anti-CRISPRs in the ESKAPE mobilome. Our results also unveiled ESKAPE-specific trends of MGEs, AMR, and virulence genes promoted by the presence of CRISPR-Cas systems. Finally, our work shows that CRISPR spacers found on prophages, ICEs/IMEs and plasmids across the ESKAPE pathogens have different targeting biases.

## MATERIALS AND METHODS

### ESKAPE pathogens collection

We retrieved all complete ESKAPE genomes available in the NCBI Reference Sequence Database (RefSeq, accessed on 12 November 2020), using ncbi-genome-download v0.3.0 (https://github.com/kblin/ncbi-genome-download). Genomes listed as ‘unverified’ were removed from our dataset. We also excluded genomes classified as ‘Enterobacteriaeceae’. Finally, we used pyANI v0.2.10 (https://github.com/widdowquinn/pyani) to calculate the average nucleotide identity based on MUMmer (ANIm) and removed genomes with an ANIm value below the 95% threshold for species delineation (45,46). To evaluate the taxonomy of the *Enterobacter* species, we retrieved genomes for *Enterobacteriaceae* type strains and used them together with the *Enterobacter* genomes to create a phylogenetic tree using GToTree v1.5.22 (https://github.com/AstrobioMike/GToTree) (47) and the IQ-TREE algorithm to estimate maximum likelihood (48). We used the pre-built set of 74 single copy gene bacterial Hidden Markov Models (HMM) available in GToTree. Genomes labelled as belonging to the *Enterobacter* genus, but clustered in the phylogenomic tree with type strains other than those from the *Enterobacter* genus, were removed from subsequent analyses. We then built a phylogenetic tree including all curated ESKAPE genomes using GToTree and the IQ-TREE algorithm as aforementioned. These trees were visualized with iTOL v6 (https://itol.embl.de/). Multi-locus sequence typing (MLST) profiles were determined with mlst v2.19.0 (https://github.com/tseemann/mlst). The curated genomes were automatically annotated using Prokka v1.14.6 (https://github.com/tseemann/prokka) (49).

### Extraction of plasmids, ICEs/IMEs and prophages

Since the ESKAPE pathogens (as most bacteria) are haploid, we separated the large replicon (i.e. the chromosome) from the extrachromosomal replicons. For the latter, only accessions with ‘plasmid’ and ‘complete sequence’ on their description were kept and were used for further plasmid analyses.

To extract ICEs from chromosomal replicons, we used the chromosomal genbank files created with Prokka as input to build a pangenome for each ESKAPE pathogen. We used ppanggolin v1.1.96 (https://github.com/labgem/PPanGGOLiN) (50), which uses a graphical model and a statistical method to partition the pangenome in persistent, shell and cloud genomes. Persistent gene families are those conserved in a large majority of genomes, while shell and cloud gene families are present at intermediate and low frequencies, respectively. We used the panRGP method to build the pangenomes (51). This method predicts regions of genome plasticity (RGPs), which are clusters of genes made of accessory genes (shell and cloud genomes) in the pangenome graph. We then used bedtools v2.30.0 (https://bedtools.readthedocs.io/en/latest/) (52) to extract RGPs from the chromosomal replicons. The proteomes of these extracted RGPs were scanned for relaxases with hmmer v.3.3.1 (http://hmmer.org/) (53) against MOBfamDB, a curated relaxase profile HMM database (54). Simultaneously, the proteomes were screened with hmmer against integrases (Pfam accession PF00589) and recombinases (PF07508). Both analyses were performed using hmmscan with default parameters. RGPs with hits both for integrases (phage integrase or recombinase) and relaxases were classified as putative ICEs/IMEs and were kept for further analysis.

To look for prophages, we masked the ICEs/IMEs locations in the chromosomal replicons using bedtools. The ICE/IME-masked chromosomes were annotated with Prokka, using as proteins of interest a collection of non-redundant viral proteins downloaded from NCBI’s RefSeq database (https://ftp.ncbi.nlm.nih.gov/refseq/release/viral/, accessed on the 25 January 2022). The masked chromosomal genbank files were then used as input in phispy v4.2.6 (https://github.com/linsalrob/PhiSpy), which combines similarity- and composition-based strategies to look for prophages (55). We also masked the prophage regions in the chromosomal replicons, to build the final masked genomes, that are ICE/IME- and prophage-masked (these masked replicons are also free of plasmids, since these are part of the extrachromosomal replicons).

### Network-based approach

To estimate the pairwise distances between all ESKAPE MGE types (i.e. plasmids, ICEs and prophages), we first ran the MMSEQseqs2 v13.45111 package (56), using 90% sequence identity for clustering each MGE type. We then reduced the dereplicated MGEs into sketches and compared the Jaccard index (JI) and mutation distances between pairs of MGEs using BinDash v 0.2.1 (https://github.com/zhaoxiaofei/bindash)(57). Each MGE sequence was converted to a set of 21-bp *k*-mers. We used the mean() and median() functions in R to calculate the arithmetic mean and median of the JI, respectively. Only JI equal to or above the mean and median were considered, and the mutation distances were used as edge attributes to plot the network with Cytoscape v3.9.0 under the prefuse force directed layout (https://cytoscape.org/). We used the Analyzer function in Cytoscape to compute a comprehensive set of topological parameters, such as the clustering coefficient, the network density, the centralization, and the heterogeneity.

### Functional annotation

COGs annotation of the MGE proteins was carried out through sequence alignments against the COGs 2020 database (https://www.ncbi.nlm.nih.gov/research/cog-project). The alignments were performed with DIAMOND v0.9.10.111 (58) with a cutoff evalue of 1e–05 and 80% coverage of both query and subject sequences. The COGs database was set up using a python script (https://github.com/kkpenn/merger_COG2020/blob/main/merger_2.py) and DIAMOND makedb with default settings. Around 36, 38 and 55% of the proteins encoded in plasmids, ICEs/IMEs, and prophages matched a protein in the COGs database, respectively, and were therefore annotated with the information of their corresponding homolog. Lists of non-redundant COG definitions (e.g. COG0105) were extracted separately for prophages, plasmids and ICEs/ IMEs, and compared with venny v2.1.0 (https://bioinfogp.cnb.csic.es/tools/venny/index.html) to identify unique and shared COGs. Likewise, COGs occurrence was determined separately for the proteomes of the three MGE types in the different ESKAPE. Information on the COGs classification into functional categories was retrieved from https://ftp.ncbi.nih.gov/pub/COG/COG2020/data/. The relative frequency of the different COG functional categories per MGE/ESKAPE pair was calculated by summing up the occurrences of COGs belonging to a given functional category and dividing the resulting number by the total number of proteins observed in the corresponding MGE/ESKAPE pair.

To explore the diversity of MGE-encoded proteins, we combined their proteomes (943246 proteins) and clustered them using the cluster algorithm from the MMseqs2 package (56). The proteins were clustered at 80% sequence identity, 80% coverage, and otherwise default settings to match the parameters used by ppanggolin when generating the ESKAPE pangenomes. The relative frequency of the different protein clusters per MGE/ESKAPE pair was calculated following the same approach used to estimate the relative frequency of COG functional categories but using the occurrence of proteins belonging to a given cluster instead. Representatives of the 72247 clusters identified were annotated with eggNOG-mapper v2 (59) with default settings to explore the functions of the MGE-encoded proteins further.

We used abricate v1.0.1 (https://github.com/tseemann/abricate) to scan extracted MGEs and masked genomes against antimicrobial resistance and virulence genes (using pre-downloaded databases from Resfinder (60) and VFDB (61) containing 3138 and 4329 sequences, respectively, and both updated on the 28 March 2022). We used default parameters, except for a 90% identity and 90% coverage thresholds. To identify and classify CRISPR-Cas systems, we used CRISPRCasTyper v1.2.3 with default thresholds for CRISPR and Cas detection (https://github.com/Russel88/CRISPRCasTyper), including a maximum of 3 unknown genes between Cas genes in the operon, an overall *E*-value threshold of 0.01, and a 10kb distance threshold to connect Cas operons and CRISPR arrays (62). We also used this tool to look for CRISPR spacers. The entire CRISPR arrays identified on MGEs were then masked using bedtools, and these masked MGEs served as a local blast database using blast v2.12.0 (https://blast.ncbi.nlm.nih.gov/Blast.cgi?PAGE_TYPE=BlastDocs), when using MGE CRISPR spacers as a query. CRISPR spacers from masked genomes were also blasted against a local database of our extracted (non-masked) MGEs. Hits with at least 95% nucleotide identity and 95% sequence coverage were considered as spacer targets (63). While a representative collection of plasmids and virus is publicly available at RefSeq's NCBI database (*n* = 33 269 and 13 778, respectively, accessed on the 21 May 2021), a substantially smaller collection of ICEs/IMEs is available at ICEberg database (*n* = 1325), and was last updated in September 2018. Due to this limitation in the number of publicly available ICEs/IMEs sequences, and to have a representative collection of these three different types of MGEs, we focused on the curated dataset presented in this study to look for targets of CRISPR spacers. Additionally, we mapped the CRISPR spacers against annotated genes across the ESKAPE mobilome, using the same blast approach and the same thresholds.

We retrieved an Anti-CRISPR collection of 1111 non-redundant proteins from Anti-CRISPRdb v2.2 (http://guolab.whu.edu.cn/anti-CRISPRdb/, accessed on the 29 March 2022). This collection was used to build a local database with DIAMOND (https://github.com/bbuchfink/diamond) (58). We used the blastp command in diamond to scan the MGEs and masked proteomes against the anti-CRISPR local database, using an identity and coverage threshold of 90%. We used an amino acid-based homology approach to find anti-CRISPRs encoded in the ESKAPE mobilome. Even though recent approaches have applied a guilt-by-association method to identify new Anti-CRISPRs (30), currently there is no tool available to apply this method in a large dataset of bacterial genomes.

### Statistical analysis

Comparisons between MGEs’ GC content and sequence length were performed using the Kruskal–Wallis test, and the *P*-values adjusted using the Holm–Bonferroni method. Comparisons between pairs of conspecific genomes with and without CRISPR-Cas systems, as well as between MGE targets for CRISPR spacers, were performed using the Wilcoxon test, and the p-values adjusted using the Holm–Bonferroni method. Values above 0.05 were considered as non-significant (ns). We used the following convention for symbols indicating statistical significance: * *P* ≤ 0.05, ** *P* ≤ 0.01, *** *P* ≤ 0.001 and **** *P* ≤ 0.0001.

## RESULTS

### MGEs are unevenly distributed among the ESKAPE pathogens

**Figure 1.**
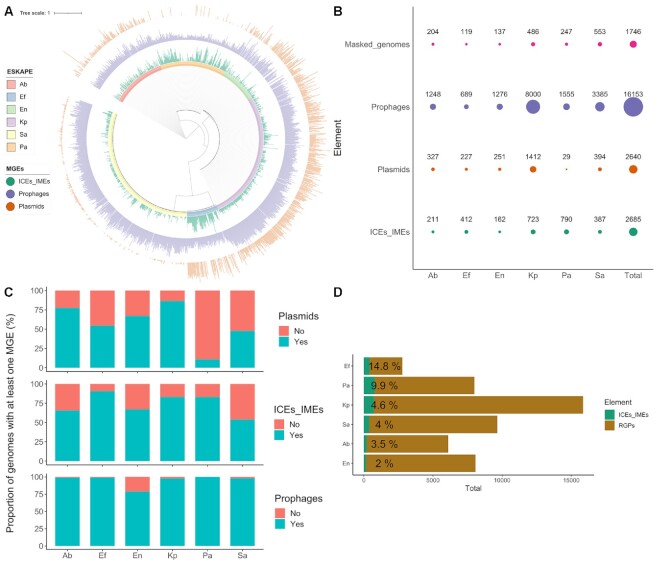
Distribution of MGEs across the ESKAPE pathogens. (**A**) Maximum likelihood tree representing the ESKAPE genomes. Tree nodes are coloured according to the ESKAPE pathogen. Three bar charts with aligned fields are shown outside the tree: the innermost bar chart shows the density of ICEs/IMEs, while the density of prophages and plasmids across the genomes are shown in the middle and outermost bar charts, respectively. (**B**) Total number of MGEs and of considered masked genomes per ESKAPE pathogen. Size of the circles is proportional to the number of identified elements. (**C**) Proportion of genomes carrying at least one plasmid, ICE/IME or prophage. (**D**) Total number of RGPs and ICEs/IMEs per ESKAPE. The size of the green bars is proportional to the total number of ICEs/IMEs identified per ESKAPE pathogen, and the relative number of ICEs/IMEs per RGPs is shown in percentage next to the green bars. Bars are sorted according to the relative number of ICEs/IMEs per RGPs. Ab, *A. baumannii*; Ef, *E. faecium*; En, *Enterobacter* sp.; Kp, *K. pneumoniae*; Pa, *P. aeruginosa*; Sa, *S. aureus*.

We downloaded 1782 ESKAPE complete genomes from NCBI’s RefSeq database. To correct for species taxonomy, genomes with <95% average nucleotide identity (ANI) were removed for each ESKAPE species (Supplementary Table S1). Since this parameter is only applied for species delineation, we also built a phylogenomic tree with *Enterobacter* sp. genomes and type strains belonging to the *Enterobacteriaceae* family (Supplementary Figure S1). Our curated dataset included 1746 complete genomes which belong to 451 different MLST profiles (Supplementary Table S2). We found a total of 21 478 MGEs, including 16 153 prophages, 2685 ICEs/IMEs and 2640 plasmids (Figure 1A and B). The density of these MGEs (i.e. the cumulative length of each MGE type per genome length) shows a patchy distribution across the ESKAPE phylogeny (Figure 1A and Supplementary Figure S2). *S. aureus* genomes are densely populated by prophages, while ICEs/IMEs are prevalent in *P. aeruginosa*. *K. pneumoniae* and *Enterobacter* are populated by plasmids and prophages. In fact, plasmids were prevalent in every ESKAPE except *P. aeruginosa* and *S. aureus* (Figures 1A and C). The majority of plasmids carried a relaxase (62.5%, 1651/2640), and were classified as mobilizable (either self-conjugative or not) (64). Curiously, *E. faecium* genomes have high densities of both prophages, plasmids and ICEs/IMEs (Figure 1A).

To look for RGPs exclusively integrated in the chromosome, we used the 1746 chromosomal replicons to generate plasmid-free pangenomes for each ESKAPE taxon. We identified a total of 50482 plasmid-free RGPs in chromosomal replicons (Figure 1D). Of these, 2685 were classified as ICEs/IMEs due to the presence of relaxase and integrase domains (Figure 1B and D). At least one ICE/IME was detected in >50% of genomes for all ESKAPE pathogens and was abundant in *E. faecium* and *P. aeruginosa* (∼3 elements/genome) (Figure 1B and C). After masking the ICEs/IMEs identified in the ESKAPE chromosomes, we performed a search for prophages. These elements were the most abundant MGE type found in the ESKAPE collection. Additionally, prophages were significantly more prevalent than ICEs/IMEs and plasmids across all ESKAPE pathogens (Supplementary Figure S2).

When looking into the presence/absence combination of co-occurring MGEs across the ESKAPE pathogens, we noticed that the most frequent combination involved the presence of the three MGEs (in 717 out of the 1746 genomes, Supplementary Table S2). We noticed that the majority of the strains with the three MGEs co-occurring in the same genome belonged to *K. pneumoniae* (340/717). Our results show that different MGEs are asymmetrically distributed across the ESKAPE pathogens, with *K. pneumoniae* genomes taking the lead for the co-occurrence of ICEs/IMEs, plasmids and prophages.

### MGE sequence similarity varies across the ESKAPE mobilome

MGEs tend to have a GC content lower than that of the remainder of its host genome (65–67). Here, we explore how conserved is this trend across different MGE types from all ESKAPE pathogens. We confirmed that for most MGE/ESKAPE pairs, the arithmetic mean GC content of the different MGEs is significantly lower when compared to masked genomes across the ESKAPE pathogens (Supplementary Figure S3A, *P*-value < 2.2e–16). With the exception of *S. aureus*, we observed that plasmids across the ESKAPE pathogens show more variation in size when compared with ICEs/IMEs and prophages (Supplementary Figure S3B). Across all ESKAPE pathogens, we observed a weak positive correlation between the ICEs/IMEs and plasmids’ GC content and sequence length (*R* = 0.38 and 0.35, respectively, *P* < 2.2e–16), and a weak negative correlation between the prophages’ GC content and sequence length (*R* = −0.15, *P* < 2.2e−16, Supplementary Figure S4). Similar Pearson correlation coefficients were observed for plasmids and prophages in a previous study (67). The underlying reasons for this correlation are unclear and warrant further research.

**Figure 2.**
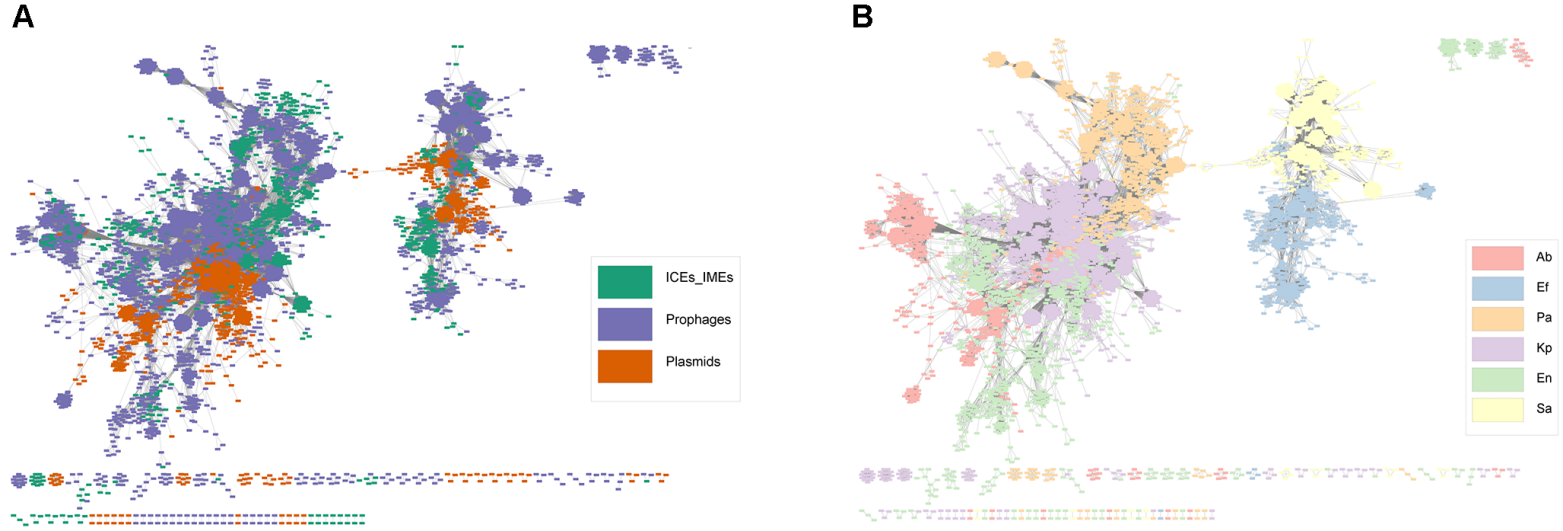
Network of clustered MGEs, using the mean Jaccard index as a threshold. Network grouped by (**A**) MGE type; and (**B**) ESKAPE pathogen. Each MGE is represented by a node, connected by edges according to the pairwise distances between all MGE pairs. The network has a clustering coefficient of 0.781, a density of 0.014, a centralization of 0.065, and a heterogeneity of 0.959. Ab, *A. baumannii*; Ef, *E. faecium*; En, *Enterobacter* sp.; Kp, *K. pneumoniae*; Pa, *P. aeruginosa*; Sa, *S. aureus*.

Given the presence of highly similar MGEs in our dataset, we dereplicated the 21 478 elements found here into a representative set of 10 339 MGEs. Each MGE was then reduced to a set of *k*-mers and the Jaccard index (JI) was used as a measure of DNA sequence similarity between all MGE pairs. The majority of MGE pairs shared little similarity, with a JI value below 0.25 (Supplementary Figure S5A), in accordance with the high diversity frequently observed across MGEs. We then used an alignment-free sequence similarity comparison of the ESKAPE mobilome to infer an undirected network (Figure 2A and B). To plot this network, we used as a threshold the mean value (0.0537361) of the estimated pairwise distances between the 10339 MGEs identified in this study (Supplementary Figure S5A). The sparse network assigned 97.8% (10110/10339) of the MGEs into 87 clusters. The network revealed clear structural differentiation, where the majority of the smaller clusters were homogeneous for a given ESKAPE/MGE pair (Figure 2A and B). The absence of pairwise distance similarities with intermediate JI (Supplementary Figure S5A) helps to explain this clustering in discrete groups, instead of a continuous genetic structure. However, the two largest clusters challenge interspecies and MGE type barriers and correspond to multiple MGEs with the four Proteobacteria representatives in the first (i.e. *K. pneumoniae*, *Enterobacter* sp., *P. aeruginosa* and *A. baumannii*), and *S. aureus* and *E. faecium* in the second cluster. MGEs within these promiscuous clusters tend to be more dissimilar than those assigned to densely connected and ESKAPE/MGE pair-restricted clusters. We observed high genetic similarity between different MGE/ESKAPE pairs (Supplementary Figure S6). We also observed cross-phylum interactions, between different MGEs across the ESKAPE pathogens. In parallel, we built a network using the masked genomes as nodes connected by edges indicating pairwise distances, and we exclusively observed species-specific clustering (Supplementary Figures S5B and S7), which is in agreement with selective forces that favor the genomic coherence of bacterial species (68,69). Altogether, our results highlight that while some elements are found in multiple genera (and phyla), for the majority of clusters host similarity and MGE type restrain DNA sharing between different ESKAPE MGEs.

### The proteome of the ESKAPE mobilome is highly diverse in sequence and functions

**Figure 3.**
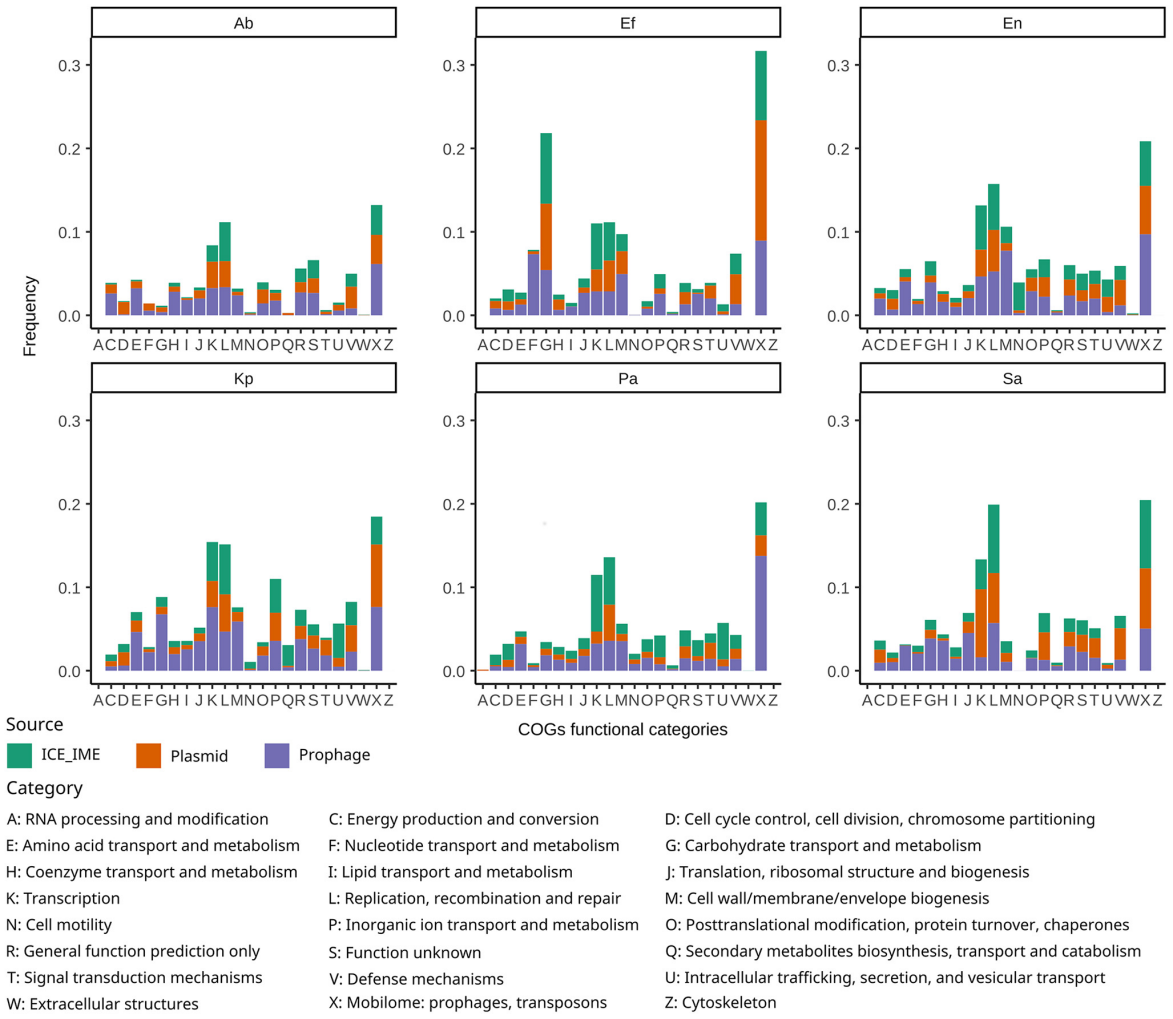
Relative contribution of ESKAPE MGEs proteome to COG functional categories. The barplots in the figure are split into facets corresponding to the different ESKAPE pathogens. The relative frequency of proteins in the different COG functional categories was calculated separately for each MGE type by dividing the number of proteins belonging to a given category by the total number of proteins observed in the corresponding MGE/ESKAPE pair. Hence, the bars illustrate the incidence of proteins of a given functional category in the proteomes of the different MGE types per ESKAPE. The COG functional categories are indicated on the X-axis and described at the bottom of the figure.

To gain functional insights into the proteome of the ESKAPE mobilome, we investigated the diversity of clusters of orthologous groups (COGs) encoded by MGEs identified in this study. COGs are protein sets conserved across lineages that typically share function and are therefore used for systematic function prediction in poorly characterised genomes (70). COGs have been assigned to curated and uniform functional categories, thus enabling the comparison of their distribution amongst genomes (71). We distinguished 2761 different COGs in the ESKAPE MGEs (Supplementary Figure S8). These clusters encompass most functional categories reported in the COGs scheme (Figure 3), thereby highlighting the diversity of functions associated with the ESKAPE mobilome. ICEs, plasmids, and prophages contain 148, 164 and 794 unique COGs, respectively, consistent with their distinctive features as mobile elements (Supplementary Figure S8). However, they also share 921 COGs (∼33%), indicative of a common pool of proteins and functions carried by MGEs in ESKAPE pathogens. COGs present in only two of the three MGE types were also identified, with prophages and plasmids sharing more COGs than other pairs (Supplementary Figure S8).

From 36% to 55% of proteins in the different MGE proteomes were assigned to a COG (see methods). Interestingly, we detected some variation in the relative contribution of MGE proteomes to different COG functional categories among the ESKAPE pathogens (Figure 3). For example, proteins associated with ‘Carbohydrate transport and metabolism’ (category G) were more frequent in MGE proteomes of *E. faecium* than in other ESKAPE (Figure 3). The relative frequency of proteins in the ‘Cell wall/membrane/envelope biogenesis’ category (M) also varied noticeably across MGE/ESKAPE pairs. On the other hand, proteins of the ‘Transcription’ and ‘Replication, recombination and repair’ categories (K and L, respectively) were among the most frequent in the MGE proteomes of all ESKAPE. As expected, proteins assigned to the COGs mobilome category (X) dominated all the MGE proteomes.

To explore the diversity of the ESKAPE MGEs proteome further, we clustered their 943 246 proteins based on sequence similarity, resulting in 72 247 groups (Supplementary Table S3). Around 69% of the representatives of these protein groups were assigned the tag ‘hypothetical protein’ by prokka, underlining the large proportion of uncharacterised proteins encoded by ESKAPE MGEs. Among the representatives with an assigned function, transposases and integrases were the most frequent protein product (2290 cluster representatives) (Supplementary Table S3). Recombinase, repressor and resistance, were also common terms across the representative products with >200 occurrences each; the latter being mostly associated with metal or drug resistance.

**Figure 4.**
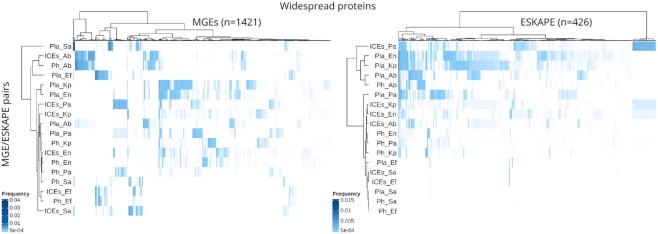
Distribution of widespread protein clusters across MGE/ESKAPE pairs. The heatmaps show the distribution and relative frequency of protein clusters (columns) identified as widespread in either MGEs (i.e. present at least once in the three MGE types; left side) or ESKAPE pathogens (i.e. present in at least three different ESKAPE; right side). The number of protein clusters represented in the heatmaps is shown in parenthesis. MGE/ESKAPE pairs are indicated on the left side of the heatmaps. The relative frequency of the protein clusters in the different MGE/ESKAPE pairs was calculated by dividing the number of occurrences identified by the total number of proteins observed in a given pair. The trees displayed at the top and left side of the heatmaps illustrate the hierarchical clustering of protein clusters and MGE/ESKAPE pairs using the ward.D method with euclidean distance. A list of the protein clusters, their products and relative frequency values is provided in Supplementary Table S3.

We then looked for protein clusters widespread in MGE proteomes, i.e. those observed in the three MGE types or in at least three of the ESKAPE pathogens. Our search resulted in the identification of 1421 protein clusters widespread across MGEs and 426 present in at least three different ESKAPE (Supplementary Table S3), with 187 clusters identified in common between the two widespread categories. Although hypothetical proteins dominated both widespread categories (55% of MGEs and 50% of ESKAPE widespread protein clusters), various protein clusters with functions associated with transposition and AMR were also identified (Supplementary Table S3). Hierarchical clustering of the MGE/ESKAPE pairs and widespread protein clusters based on the distribution and relative frequency of the latter uncovered structured patterns of sharing (Figure 4). For example, we detected a component of ESKAPE-widespread protein clusters present in plasmids of *Enterobacter* sp., *K. pneumoniae* and ICEs/IMEs of *P. aeruginosa*. When it comes to protein clusters present in different MGE types, we observed clusters predominantly occurring in ICEs/IMEs and phages of *A. baumannii* and *E. faecium*. Overall, the distribution of ESKAPE widespread proteins clustered MGE/ESKAPE pairs by MGE type (Figure 4). The clustering observed from the distribution of MGE widespread proteins was more intricate, with only a couple of clusters featuring the same ESKAPE pathogen. Altogether, our results show that more than seventy thousand protein clusters, representing nearly one million sequences, are linked to the mobilome component of the ESKAPE pangenomes. These proteins are involved in a broad range of functional categories; frequently in transcription, replication and recombination. Only ∼2.3% of protein clusters are widespread within the ESKAPE MGEs context, but they feature complex distribution and frequency patterns.

### AMR genes are overrepresented in the ESKAPE mobilome

In order to explore the AMR repertoire of the ESKAPE mobilome, we only focused on genes that are horizontally transferred, such as beta-lactamases and aminoglycoside-modifying enzymes (that lead to antimicrobial inactivation), and those that promote target site modification (such as rRNA methyltransferases, the *vanA* and *vanB* gene clusters, and the staphylococcal cassette chromosome *mec*). We observed that AMR genes are broadly distributed in plasmids across the ESKAPE pathogens. Even though the total number of prophages far outnumber that of plasmids in our collection (Figure 1), the absolute count of AMR genes in plasmids is greater than that observed in prophages (6068 versus 1845, respectively) (Supplementary Figure S9). Interestingly, most AMR genes in plasmids and prophages are found in *K. pneumoniae*, whereas *P. aeruginosa* carries the majority of these genes within ICEs/IMEs (Supplementary Table S4). All ESKAPE pathogens have a large proportion of AMR-carrying plasmids (>35% of plasmids across the different ESKAPE carry at least one AMR gene), while a high proportion of AMR-harbouring ICEs (>25%) was only observed for *S. aureus* and *P. aeruginosa* (Supplementary Figures S10A and B). As previously reported (72), we observed that AMR genes are rarely found in prophages (<12% of prophages across the different ESKAPE carry at least one AMR gene) (Supplementary Figure S10C). As expected from the vast repertoire of MGEs present in *K. pneumoniae* (Figure 1A), this species presented a wider selection of different AMR genes. Some AMR genes were restricted to specific ESKAPE pathogens, while others were more promiscuous. For example, different beta-lactamases (*bla* genes) were prevalent among proteobacterial representatives of the ESKAPE pathogens but were mostly absent from *S. aureus* and *E. faecium* (Supplementary Figure S9 and Table S4). The only exception was the *blaZ* gene, which was exclusively identified in plasmids, prophages, and ICEs/IMEs from *S. aureus*. This gene is typically embedded within the SCCmec elements of this species and may have been acquired from distantly related non-*Staphylococcus* species (73). Genes encoding resistance to aminoglycosides (*aac*, *ant* and *aph* genes), chloramphenicol (*cat* genes), trimethoprim (*dfr* genes) and tetracyclines (*tet* genes) were found in all representatives of the ESKAPE pathogens. Genes involved in resistance to vancomycin (the *vanHAX* and *vanHBX* gene clusters) were exclusively found in *S. aureus* and *E. faecium*, while genes coding resistance for quinolones (*qnr* genes) and colistin (*mcr* genes) were only found in the proteobacterial representatives (Supplementary Figure S9 and Table S4).

We next assessed the distribution of virulence genes. These genes are broadly distributed in prophages and ICEs/IMEs across the ESKAPE pathogens (Supplementary Figure S11 and Table S5). In fact, we identified no virulence genes in *E. faecium* plasmids, and only 0.6% of *A. baumannii* plasmids carry these genes. More than 25% ICEs/IMEs in *S. aureus*, *K. pneumoniae*, and *E. faecium* carried at least one virulence gene (Supplementary Figures S10C and D). *P. aeruginosa* is the ESKAPE pathogen carrying a wider variety of virulence genes in these MGEs, mostly on prophages. Polyketide synthesis loci *ybt* and *clb* encoding the iron-scavenging siderophore yersiniabactin and genotoxin colibactin, respectively, were solely identified in *Enterobacteriaceae* representatives of the ESKAPE pathogens (i.e. *K. pneumoniae* and *Enterobacter* sp.). These virulence loci were mostly present in ICEs/IMEs, as previously reported (74), but we also found these genes on plasmids and prophages (Supplementary Figure S11 and Table S5). Interestingly, *S. aureus* was the ESKAPE pathogen with a higher proportion of both plasmids and ICEs/IMEs carrying at least one AMR or virulence genes (Supplementary Figure S10).

**Figure 5.**
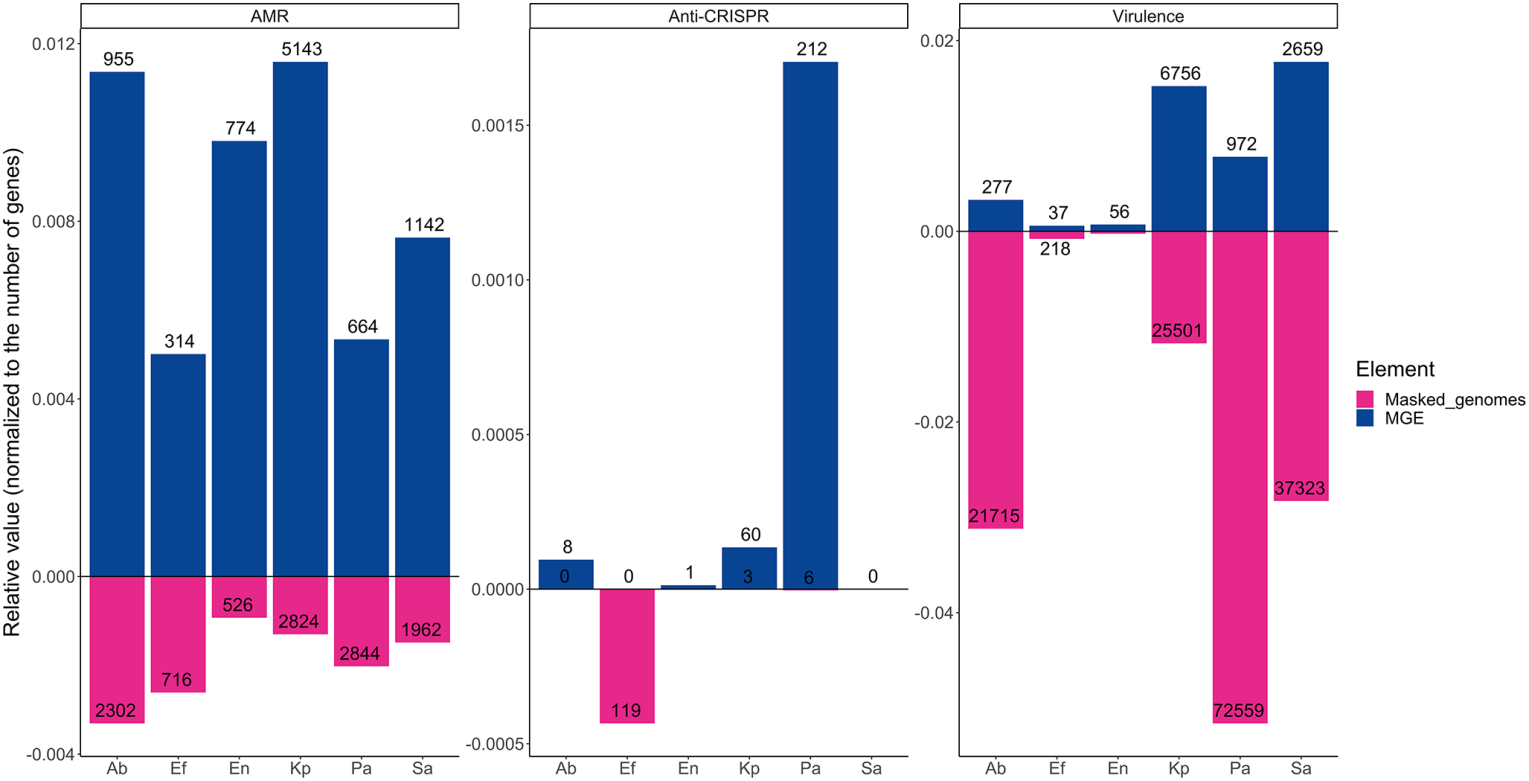
Relative proportion of AMR, virulence, and Anti-CRISPRs between MGEs and masked genomes across the ESKAPE pathogens. The number of AMR, virulence, or Anti-CRISPRs proteins found for MGEs or masked genomes per ESKAPE was normalized to the total number of proteins found for each element per ESKAPE pathogen. Absolute counts of AMR, virulence or anti-CRISPRs proteins is shown inside or outside the bars.

Since chromosomes are substantially larger than MGEs and consequentially have more genes, we corrected the prevalence of AMR and virulence genes to the total number of genes present in MGEs and masked genomes across the different ESKAPE pathogens. Overall, we noticed that AMR genes were largely overrepresented in MGEs (∼5×), when compared with masked genomes (Figure 5). On the other hand, virulence genes were ∼2× more likely to be located on masked genomes. Taken together, our results show that plasmids are important vectors for AMR genes across the ESKAPE pathogens, while ICEs/IMEs and prophages play a more important role for the distribution of virulence genes. When compared with masked genomes, we confirmed that AMR genes are preferentially distributed in the ESKAPE mobilome.

### CRISPR-Cas systems shape the number of MGEs, AMR and virulence genes across ESKAPE pathogens

**Figure 6.**
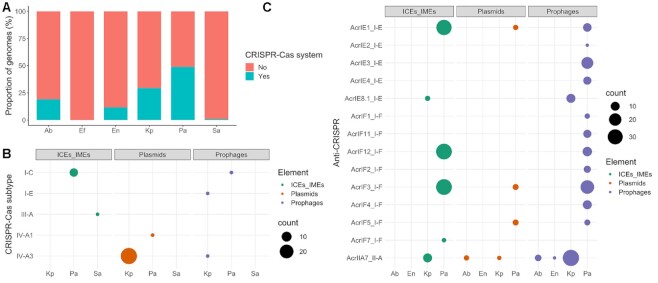
Distribution of CRISPR-Cas and Anti-CRISPR across the ESKAPE pathogens. (**A**) Proportion of CRISPR-Cas positive genomes across the ESKAPE pathogens. (**B**) Distribution of CRISPR-Cas systems within ICEs/IMEs, plasmids, and prophages. (**C**) Distribution of Anti-CRISPR proteins across ICEs/IMEs, plasmids, and prophages. Even though we found anti-CRISPRs on masked genomes, we only plotted those found on MGEs. A complete list of Anti-CRISPRs is given in Supplementary Table S8. Ab, *A. baumannii*; Ef, *E. faecium*; En, Enterobacter sp.; Kp, *K. pneumoniae*; Pa, *P. aeruginosa*; Sa, *S. aureus*.

We focused on CRISPR-Cas systems as an example of a bacterial defense system. CRISPR-Cas systems were identified in every ESKAPE pathogen except *E. faecium*, which was therefore excluded from subsequent analyses. The proportion of genomes with CRISPR-Cas systems varied across the ESKAPE pathogens, from around 45.7% for *P. aeruginosa* to around 0.7% for *S. aureus* (Figure 6A and Supplementary Table S2). We then explored the prevalence of CRISPR-Cas systems across closely related strains belonging to the same MLST profile. Since *Enterobacter* sp. consists of multiple species, this ESKAPE pathogen was excluded from subsequent analyses. Given the low prevalence of CRISPR-Cas systems in *S. aureus*, this species was also excluded, and we focused exclusively on *P. aeruginosa*, *K. pneumoniae*, and *A. baumannii*. Interestingly, some sequence types (ST) consisted entirely of either CRISPR-Cas positive or negative genomes (Supplementary Figure S12 and Table S2). For example, the most frequent MLST profile in *A. baumannii* from our dataset was ST2 (*n* = 101), which only included strains with no CRISPR-Cas systems. On the other hand, the second most prevalent MLST profile in this species (ST1, *n* = 14), only consisted of strains with I-F CRISPR-Cas systems. The most frequently observed MLST profile from *K. pneumoniae* (ST11, *n* = 105), consists mostly of CRISPR-Cas negative strains (96.2%, 101/105). The four strains with positive hits carried IV-A3 CRISPR-Cas systems on plasmids. ST258 (*n* = 47) was the second most common *K. pneumoniae* MLST profile identified in our dataset, and again, consisted entirely of strains with no CRISPR-Cas systems. However, ST147 and ST15 (*n* = 31 and *n* = 23, respectively) carried I-E CRISPR-Cas systems in all strains. Finally, looking at *P. aeruginosa*, we found that the most prevalent MLST profiles in our dataset (ST235 and ST549, *n* = 16 and *n* = 11, respectively) carried no CRISPR-Cas systems. The only exception was found in a ST235 strain, which carried an I-C CRISPR-Cas system on an ICE/IME. In contrast, ST233 and ST1971 (*n* = 8 for both) consisted exclusively of strains carrying the I-F CRISPR-Cas system on masked genomes (Supplementary Table S2). These findings suggest that the presence or absence of CRISPR-Cas systems across the ESKAPE pathogens is related to sequence type and thus most likely due to phylogenetic history of the strains (Supplementary Figure S12).

Our analysis revealed a large variety of MGE-encoded CRISPR-Cas subtypes, with I-C, I-E, III-A, IV-A1 and IV-A3 represented across the dataset (Figure 6B). We found CRISPR-Cas systems on plasmids (*n* = 28 IV-A3 subtype in *K. pneumoniae* and *n* = 1 IV-A1 in *P. aeruginosa*), ICEs/IMEs (*n* = 7 I-C in *P. aeruginosa* and *n* = 1 III-A in *S. aureus*), and prophages (*n* = 1 I-C in *P. aeruginosa*, *n* = 1 IV-A3 and *n* = 1 I-E both in *K. pneumoniae*) (Figure 6B and Supplementary Table S6). The plasmids and ICEs/IMEs carrying these systems were large, ranging from 102 to 430 kb. We also observed that all CRISPR-Cas-carrying plasmids have a MOB relaxase. This is in agreement with previous findings (75), which reported an enrichment of CRISPR-Cas systems across plasmids with conjugative functions and of larger sizes. We also found AMR and virulence genes on these CRISPR-Cas positive MGEs, but no anti-CRISPRs within the boundaries of these MGEs, suggesting that the CRISPR-Cas systems are functional.

When looking into the influence of GC content and sequence length in pairs of conspecific ESKAPE pathogens with and without CRISPR-Cas systems, we would expect to observe smaller and GC richer strains on those carrying these systems. Size expectations could only be met for *P. aeruginosa*, for which CRISPR-Cas positive genomes were significantly smaller than their counterparts (Supplementary Figure S13A, *P*-value 0.0028), as observed before (76–78). Surprisingly in *K. pneumoniae*, genomes with CRISPR-Cas systems were significantly larger than CRISPR-Cas negative genomes (Supplementary Figure S13A, *P*-value 0.0023). The non-significant differences observed for *A. baumannii*, *Enterobacter* sp. and *S. aureus* could in part be explained by the low sample size of CRISPR-Cas positive genomes (Figure 6A). Regarding the GC content, we observed significant differences in *A. baumannii*, *K. pneumoniae*, and *P. aeruginosa*. CRISPR-Cas positive genomes were GC richer in *A. baumannii* and *P. aeruginosa* (Supplementary Figure S13B, *P*-values 0.0013 and 0.046, respectively). Curiously, we noticed that CRISPR-Cas positive genomes in *K. pneumoniae* were GC poorer (Supplementary Figure S13B, *P*-value 5.7e−09). Given the known association between GC content and genome size (67), these GC differences in CRISPR-Cas positive and negative *P. aeruginosa* genomes may be a spurious correlation driven by small size of CRISPR-Cas positive genomes. So, we corrected the GC content for variation in genome size, and we observed that the association was maintained (Supplementary Figure S14A, *P*-value 0.0035), in accordance to a previous study (77).

Since virulence genes are overrepresented in the chromosome (Figure 5), we assessed the distribution of these genes in pairs of conspecific ESKAPE pathogens with and without CRISPR-Cas systems. Virulence genes were significantly less abundant in CRISPR-Cas positive genomes from *P. aeruginosa* and *A. baumannii* (Supplementary Figure S13C, *P*-values 4.1e−06 and 0.0016, respectively). Given that *P. aeruginosa* genomes positive for these systems are significantly smaller than their CRISPR-Cas negative counterparts (Supplementary Figure S13A), the lower prevalence of these genes in CRISPR-Cas positive *P. aeruginosa* genomes may again be driven by a spurious correlation. As so, we corrected the number of virulence genes for variation in genome size, and we observed that indeed the difference was no longer significant (Supplementary Figure S14B, *P*-value 0.74), confirming our prediction that the genome size was a confounding variable obscuring the effect of CRISPR-Cas systems on the prevalence of virulence genes in *P. aeruginosa*.

**Figure 7.**
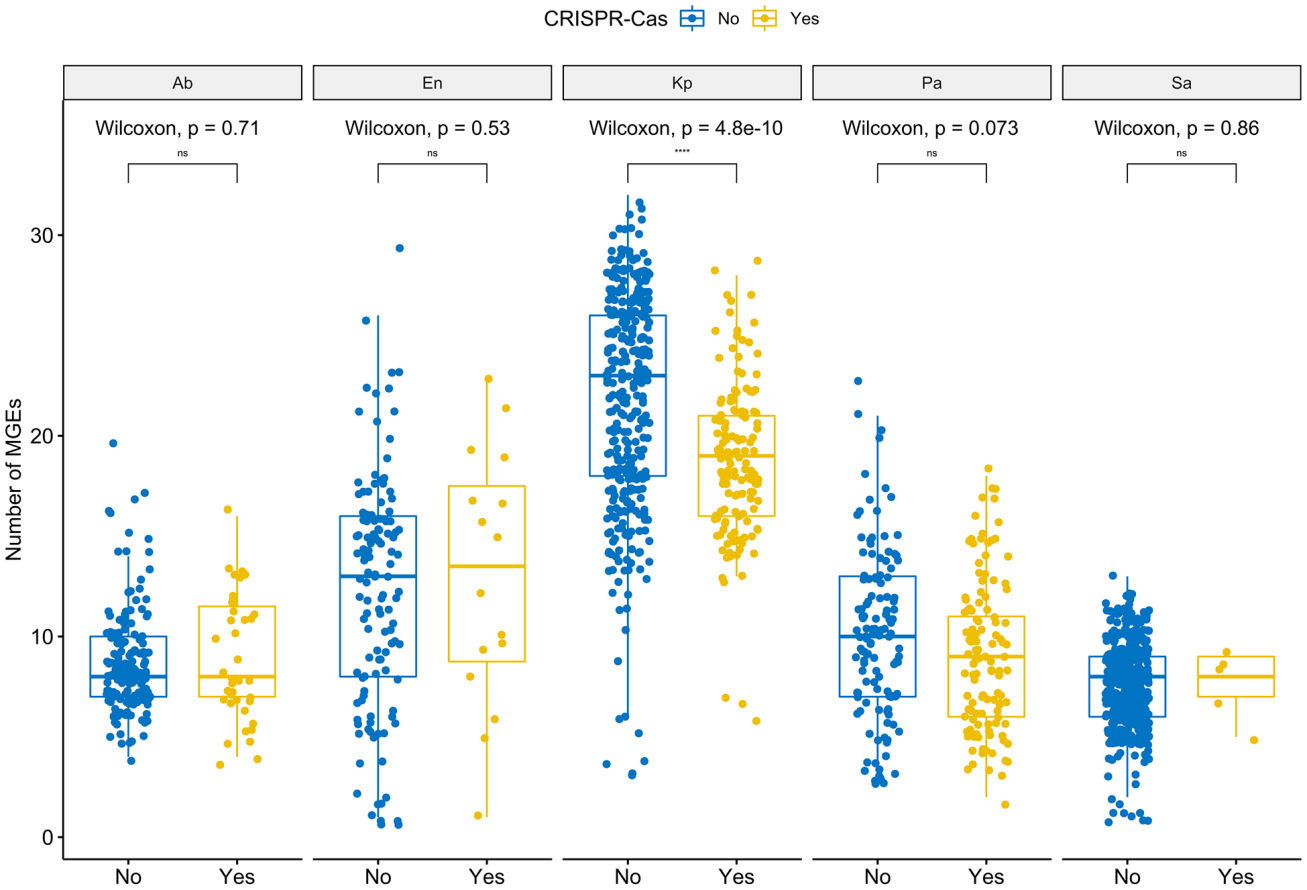
The absence of CRISPR-Cas systems does not associate with MGE increases in ESKAPE pathogens with the exception of *K. pneumoniae*. Boxplots compare the number of MGEs present in pairs of conspecific ESKAPE pathogens, with and without CRISPR-Cas systems. The graph shows the complete data distributions (individual points) and a summary of data distributions based on boxplots, where the middle horizontal line indicates the median, the boxes the quartiles above and below the median, and the whiskers the remaining quartiles. Values above 0.05 were considered as non-significant (ns). We used the following convention for symbols indicating statistical significance: * *P* ≤ 0.05, ** *P* ≤ 0.01, *** *P* ≤ 0.001 and **** for *P* ≤ 0.0001. Ab, *A. baumannii*; En, *Enterobacter* sp.; Kp, *K. pneumoniae*; Pa, *P. aeruginosa*; Sa, *S. aureus*.

We then explored whether CRISPR-Cas presence or absence reduced the number of MGEs acquired in pairs of conspecific ESKAPE pathogens. We would expect to detect a smaller number of MGEs in genomes harbouring these immune systems. However, this trend was only observed in *K. pneumoniae* (Figure 7). The variation in the number of MGEs in genomes either with or without CRISPR-Cas systems still holds when correcting for genome size (Supplementary Figure S15). Finally, we focused on AMR and virulence genes carried exclusively by plasmids and ICEs/IMEs, as these were the most important vectors (Supplementary Figure S10). For most MGE/ESKAPE pairs, we observed no significant difference between pairs of conspecific genomes with and without CRISPR-Cas systems. When it comes to AMR genes, we only observed significant differences in MGEs from *P. aeruginosa* (Supplementary Figure S16A, *P*-values 0.037). Indeed, AMR genes were more prevalent on ICEs/IMEs from *P. aeruginosa* genomes with CRISPR-Cas systems (Supplementary Table S7). A similar correlation was previously reported (78). Curiously, the less prevalent I-C CRISPR-Cas subtype, which was exclusively identified in *P. aeruginosa* and mostly on ICEs/IMEs (Supplementary Table S6), was recently found to be positively correlated with certain AMR genes (76). Regarding virulence genes, we observed significant differences in MGEs from *K. pneumoniae*, where CRISPR-Cas-carrying elements were associated with more virulence genes than their CRISPR-Cas negative counterparts (Supplementary Figure S16B, *P*-values 0.0054). Taken together, we observed species-specific trends shaping the number of MGEs, AMR and virulence genes across pairs of conspecific ESKAPE genomes with and without CRISPR-Cas systems.

### Anti-CRISPRs are overrepresented in the ESKAPE mobilome

Anti-CRISPR proteins (*n* = 410) antagonising CRISPR-Cas subtypes I-E, I-F and II-A were identified across prophages, ICEs/IMEs and prophages from all ESKAPE pathogens except *S. aureus* (Figure 6C and Supplementary Table S8). The majority of these counter-defense systems were found in prophages and ICEs/IMEs from *P. aeruginosa*. We also looked for these proteins across the masked genomes and found hits in all ESKAPE except *A. baumannii* and *S. aureus* (Supplementary Table S8). After correcting their prevalence to total number of genes, we verified that anti-CRISPRs are largely overrepresented in MGEs (∼15×) when compared with masked genomes (Figure 5). When compared with masked genomes, our results show that Anti-CRISPR proteins are preferentially encoded in MGEs.

### CRISPR spacers in ICEs/IMEs, prophages and plasmids have different targeting biases

We explored the targets for all CRISPR spacers, retrieved from complete CRISPR-Cas systems, but also orphan CRISPRs, identified in our collection of ESKAPE genomes. Since we provided here a representative dataset of prophages, ICEs/IMEs, and plasmids (*n* = 16 153, *n* = 2685 and *n* = 2640, respectively), we used this collection as a database and took the CRISPR spacers identified in masked genomes as a query. In parallel, we used the CRISPR spacers identified in MGEs as a query and the MGEs collection as a database. For the latter, and to avoid self-targeting hits, we masked all CRISPR spacers from the MGEs collection used as database. We observed that only a small fraction of MGEs carry CRISPR spacers (1.3%, 33/2640 plasmids; 0.6% ICEs/IMEs, and 0.07% prophages). A total of 1087 spacers was found across all MGEs (*n* = 554 on plasmids, *n* = 343 on ICEs/IMEs and *n* = 190 on prophages). Given the large number of MGEs and CRISPR-Cas-encoding plasmids in *K. pneumoniae* (Figures 1 and 6B), it was no surprise to observe that more than half of the spacers were found in this species (577/1087). The large majority of plasmid spacers were identified on mobilizable plasmids (99.4%, 551/554). We then corrected the total length of CRISPR spacers found on each MGE by the size of the corresponding MGE. Interestingly, we found that the density of CRISPR arrays is significantly higher across prophages than that of plasmids and ICEs/IMEs (*P*-value 3.7e−07, Supplementary Figure S17).

**Figure 8.**
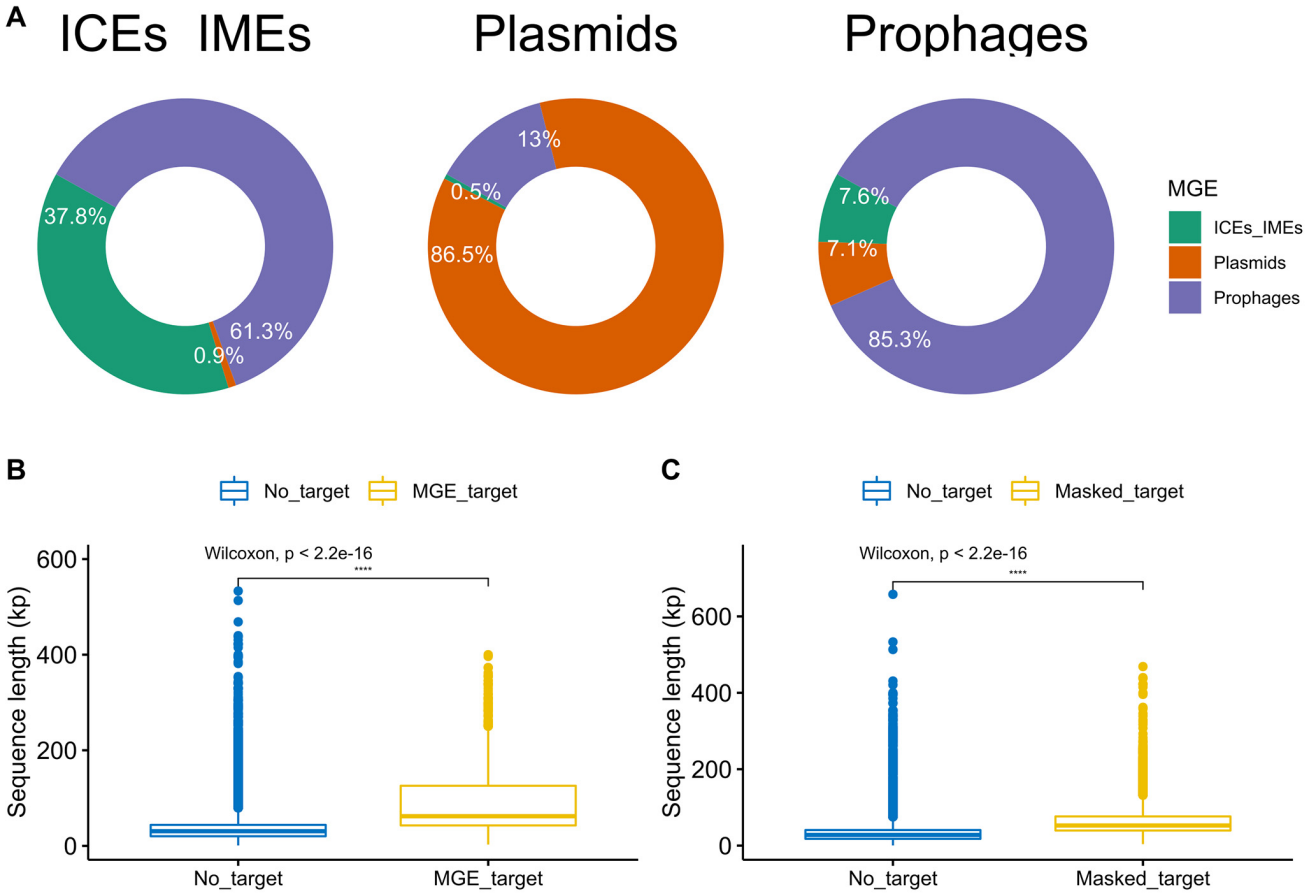
CRISPR spacers are involved in MGE-MGE conflict. (**A**) CRISPR spacers found on ICEs/IMEs (top left), plasmids (top middle) and prophages (top right), and their interactions with spacer targets identified in ESKAPE MGEs. (**B**) Significant variation in the sequence length of MGEs either targeted or not targeted by CRISPR spacers found in MGEs. (**C**) Significant variation in the sequence length of MGEs either targeted or not targeted by CRISPR spacers found in masked genomes. Graphs in (B) and (C) show a summary of data distributions based on boxplots, where the middle horizontal line indicates the median, the boxes the quartiles above and below the median, and the whiskers the remaining quartiles. Values above 0.05 were considered as non-significant (ns). We used the following convention for symbols indicating statistical significance: * *P* ≤ 0.05, ** *P* ≤ 0.01, *** *P* ≤ 0.001 and **** *P* ≤ 0.0001. Ab, *A. baumannii*; En, *Enterobacter* sp.; Kp, *K. pneumoniae*; Pa, *P. aeruginosa*; Sa, *S. aureus*.

We then looked for MGE spacer targets and identified matches for 1271 MGEs from our collection (5.9%, *n* = 1271/21 478, Supplementary Table S9). A substantially larger fraction of CRISPR spacers from plasmids targeted mobilizable plasmids from our ESKAPE collection (81.8%, 21 628/26 438 of total plasmid spacer's interactions). Only a small fraction of plasmid spacers targeted prophages (13.0%), non-transmissible plasmids (4.7%), and ICEs/IMEs (0.5%) (Figure 8A). Most prophages spacers targeted other prophages (85.3%, 1513/1773 of total prophage spacer's interactions). Only a small fraction of prophage spacers targeted ICEs/IMEs (7.6%) and plasmids (7.1%). Surprisingly and unlike CRISPR spacers found on plasmids and prophages, ICE/IMEsspacers were not biased towards other ICEs/IMEs (37.8%, 795/2102 of total ICEs/IMEs interactions), but towards prophages (61.3%). Only a small fraction of ICE/IME spacers targeted plasmids (0.9%) (Figure 8A). Most genes that are targeted by CRISPR spacers encode for hypothetical proteins (Supplementary Table S10). Still, we found that CRISPR spacers in prophages from *P. aeruginosa* can target genes encoding partition proteins on plasmids from the same species, and that the CRISPR spacers on plasmids from *K. pneumoniae* and prophages from *P. aeruginosa* target genes involved in the conjugation apparatus of plasmids from multiple ESKAPE pathogens. We also observed that CRISPR spacers from prophages and plasmids in *K. pneumoniae* target genes on ICEs/IMEs encoding lysozymes, recombinases and genes involved in conjugation. Multiple genes were targeted by CRISPR spacers on prophages from multiple ESKAPE pathogens, including those coding for portal, tail, and virion structural proteins (Supplementary Table S10). Finally, when blasting MGE spacers against the 1271 MGEs from our collection, we found no targets for 41.1% of ICE/IME spacers (141/343), 12.4% of plasmid spacers (69/554) and 0.5% of prophage spacers (1/190).

When it comes to CRISPR arrays on masked genomes, we observed a total of 13 400 spacers in 30.4% ESKAPE genomes (*n* = 531/1746). Across the *S. aureus* genomes, we found a total of 174 CRISPR spacers (31.5%, 174/553) and only four Cas operons, which explains the low prevalence of CRISPR-Cas systems in this species (<1%, Figure 6A). Consistent with the absence of CRISPR-Cas systems in the *E. faecium* strains from our dataset, no spacers were identified in masked genomes from this species. The number of CRISPR spacers per genome varied considerably between the masked genomes of the ESKAPE pathogens (Supplementary Figure S18), reaching as high as 189 in *A. baumannii*. In fact, only strains from this species carried >100 spacers per masked genome. We observed that 38.4% (5141/13 400) of CRISPR spacers in ESKAPE masked genomes yielded matches to spacer targets in MGE sequences, found on 16.4% MGEs (*n* = 3523/21 478, Supplementary Table S11). Most CRISPR spacers from masked genomes targeted prophages (44.5%, 83034/18 6619 total interactions), mobilizable plasmids (39.9%) and ICEs/IMEs (10.5%). As observed for CRISPR spacers in MGEs, CRISPR spacers from masked genomes rarely targeted non-transmissible plasmids (5.2%). We then tested if MGE or masked genome spacers preferentially targeted ESKAPE MGEs of variable size. We found that the CRISPR spacers from both MGEs and masked genomes targeted significantly larger MGEs than those with no spacer targets (*P*-value < 2.2e−16, Figure 8B and C). Altogether, our results show that plasmids and prophages mostly targeted other plasmids and prophages, respectively, while ICEs/IMEs preferentially targeted prophages. Our data also shows that CRISPR spacers found either on MGEs or masked genomes consistently target larger MGEs.

## DISCUSSION

In this work, we performed a systematic analysis of prophages, ICEs/IMEs, and plasmids across all ESKAPE pathogens. We focused on this panel because the ESKAPE group of pathogens consists of clinically-relevant bacteria, for which many genomes are completely sequenced (an important parameter when delineating MGEs), and which include representatives of both Proteobacteria and Firmicutes, and also phylogenetically divergent bacteria (with exception of *K. pneumoniae* and *Enterobacter* sp., the remaining representatives belong to different bacterial families). Studying MGEs in parallel allowed us to explore their uneven distribution across a collection of complete genomes from important pathogens, and to explore potential DNA sharing events between different MGE types. By separating these elements from masked (MGE-free) genomes, we were able to observe an overrepresentation of AMR genes and anti-CRISPRs across the ESKAPE mobilome. Furthermore, we focused on CRISPR-Cas as an example of bacterial defense systems, which are efficient against invading DNA and provide information on their targets through analysis of the associated spacer sequences (22,25), and therefore yield insight into the presence of possible defenses against the here studied MGEs. We assessed the influence of CRISPR-Cas defense systems in shaping the acquisition of MGEs and beneficial genes, and we unveiled different targeting biases for CRISPR spacers identified in plasmids, prophages and ICEs/IMEs.

The network-based approach used here to study the ESKAPE MGEs revealed a clear structural differentiation, where the majority of clusters were homogeneous for a given ESKAPE/MGE pair. Using pairwise genetic distances of alignment-free *k*-mer sequences has circumvented the exclusion of non-coding elements that was observed in gene content similarity networks from previous work (79), providing a more comprehensive picture of plasmid population and dynamics (13). Other groups have shown that plasmids form coherent clusters (13,14), similar in concept to what was observed for bacterial genomes (46,68,69). Focusing on the ESKAPE pathogens, we demonstrated here that the same happens for ICEs/IMEs and prophages for the majority of the clusters. However, two large heterogeneous clusters were also observed. Unlike bacterial genomes, where recombination between closely related replicons is the main force promoting genomic cohesiveness (46,68) (Supplementary Figure S7), MGEs such as plasmids, ICEs/IMEs and prophages are found in multiple genera (and phyla) (80). Based on our network-based approach, we observed that the ESKAPE mobilome appears to follow a bipartite structure, with some elements being capable of shuffling DNA between distantly related species.

The abundance of MGEs is strongly associated with the prevalence of AMR genes. This is particularly evident for *K. pneumoniae*, which carries a high proportion of important vectors of AMR genes, such as plasmids and ICEs/IMEs. After correcting the prevalence to total number of genes, we observed that AMR genes are nearly 5 times more likely to be found on ESKAPE MGEs than on masked genomes. Most likely the use of different antibiotics targeting either Gram negative or positive infections may have selected for the emergence of different AMR genes across the Proteobacteria/Firmicutes divide. For example, vancomycin is used as last resort for the treatment of sepsis and other infections caused by Gram-positive bacteria, while colistin mainly serves to target multi-drug resistant Gram-negative infections (31). Anti-CRISPRs are nearly 15 times more abundant on the ESKAPE MGEs than on masked genomes. Unlike AMR genes, where genes conferring resistance to similar antibiotics were identified in different ESKAPE pathogens, the distribution of virulence genes across the ESKAPE mobilome was mostly species-specific. This may be explained by different mechanisms of virulence and toxicity across bacterial species. Even considering the relative proportion of these genes, we confirmed that these genes are twice more likely to be identified in masked genomes than in MGEs.

Curiously, we found no CRISPR-Cas systems in our curated *E. faecium* dataset. In addition to our main analysis, we specifically searched for the presence of these systems on the excluded genomes, based on <95% genome similarity threshold defined for species delineation (46), and found three strains with these systems, all belonging to the II-A subtype (Supplementary Table S12). Since the majority of the most representative MLST profiles in our collection consists of genomes either with or without CRISPR-Cas systems (Supplementary Table S2), analysis of intra-ST CRISPR variability between pairs of conspecific ESKAPE genomes was not performed in this study. Except for *P. aeruginosa* (76,77), we found no evidence for genome length as a marker for HGT inhibition by CRISPR-Cas systems. A similar observation was made before, for a different collection of bacterial pathogens (78).

Defense systems such as CRISPR-Cas are inherently costly to bacterial hosts, mainly due to different forms of autoimmunity (81). To offset the short-term deleterious effects, these systems benefit from associating with MGEs, such as the examples observed here and elsewhere (42). Although these and other defense mechanisms such as restriction-modification systems do not qualify as bona fide MGEs (the systems lack mechanisms controlling their own replication), these quasi-autonomous systems take advantage of MGEs to promote their own dissemination and maintenance across bacterial hosts. Conversely, MGEs benefit from these systems and may pervasively repurpose them for inter-MGE competition (75). In fact, we found that spacers in the ICE/IME, prophage, and plasmid CRISPR arrays target competing MGEs, underscoring the genetic independence of CRISPR-Cas systems in MGE-MGE conflicts. Importantly, the presence of CRISPR-Cas subtypes preferentially distributed in MGEs (I-C and IV-A3) points to the existence of distinct selective pressure that promote the maintenance of specific subtypes on ICEs/IMEs and plasmids versus masked genomes. Given the large sizes of CRISPR-Cas systems, we observed a bias in their distribution towards larger MGEs (both plasmids and ICEs/IMEs > 100 kb). Since plasmids with an identifiable relaxase (hence classified as conjugative or mobilizable) are larger than the so-called non-transmissible plasmids (64), we unsurprisingly found a relaxase in all CRISPR-Cas positive plasmids. Prophages seem to follow similar streamlining dynamics (82). Even though nearly half of *P. aeruginosa* genomes carry at least one CRISPR-Cas system (Figure 6A and Supplementary Table S2), multiple anti-CRISPRs were found across prophages and ICEs/IMEs (Figure 6C), suggesting a potential role played by these elements in silencing immune systems in this species.

Our results shows that only a restricted fraction of CRISPR spacers matched spacer MGE targets, which is in agreement with previous findings (75,77). While the large majority of plasmid- and prophage-encoded spacers were predicted to target other plasmids and prophages, respectively, CRISPR arrays in ICEs/IMEs preferentially targeted prophages, but also a large proportion of other ICEs/IMEs (Figure 8). This complementary targeting preference can be explained by the different lifestyle of these MGEs. Since plasmids are maintained as extra-chromosomal elements, and ICEs/IMEs and prophages are integrated in the chromosome, we hypothesize that while plasmids preferentially target plasmid competitors (42), ICEs/IMEs exploit CRISPR-Cas systems to protect their host against viral predators and other ICEs/IMEs.

Several bioinformatic tools exist to look for plasmids and prophages, but currently the options for ICEs/IMEs are scarce (83). We provide here an accurate identification of these elements, building upon a recently reported tool to scan RGPs (51). However, our approach depends on the availability of a pangenome for the considered taxa, which is an important limitation for species with an insufficient number of completely sequenced genomes. It would be interesting in the future to assess the distribution of functional and non-functional prophages (and also other types of MGEs). Even though it is not always straightforward to distinguish functional from non-functional prophages, size variation could be a useful indicator. Since CRISPR arrays consist of a memory bank that is well suited to provide biological and ecological insights, and many spacer sequences can be traced back to their original locations, studying these systems yields valuable insights into the possible selective advantages of these defense systems. Nevertheless, bacteria and different MGEs employ multiple defense systems (84), next to CRISPR-Cas, and these defense systems could also influence MGE distributions. Their analysis may represent a promising focus for future research and could further help to understand limitations to the spread of MGEs.

Moreover, our work only focused on three MGE types, which are likely to be of main importance for the dissemination of genes involved in pathogenesis and AMR and thus critical for our understanding of the evolution of the ESKAPE pathogens. Nevertheless, other MGEs should be considered in future work, for example those that contribute to communication between cells (such as phage-inducible chromosomal islands (85)), or intracellular transfer events (e.g. transposons and insertions sequences (19)).

To conclude, our results indicate that prophages, ICEs/IMEs, and plasmids are asymmetrically distributed across the ESKAPE pathogens. We found that these MGEs can be found in multiple genera (and phyla), even though most clusters are constrained by host similarity and/or the type of MGE. We observed that the proteome of ESKAPE MGEs is highly diverse, involved in diverse functional categories, and features convoluted distribution patterns (including both MGE/ESKAPE specific and widespread proteins). When comparing against masked (MGE-free) genomes, we observed the pervasive association of AMR genes and anti-CRISPRs with the ESKAPE mobilome. We also found different targeting biases to CRISPR spacers found on plasmids, prophages, and ICEs/IMEs, highlighting their genetic independence. Taken together, our results illustrate the potential of network-based approaches and comparative genomics to underscore the composition and dynamics of gene flow across different MGEs, and sheds a new light in the role of the overlooked ICEs/IMEs as important players in the MGE-MGE warfare in the ESKAPE pathogens and thus the main groups of highly problematic human pathogens.

## DATA AVAILABILITY

Analyses were made with a combination of shell and R 4.0.3 scripting. R scripts and supporting tables used to create the figures are available at https://gitlab.gwdg.de/botelho/eskape_paper).

## Supplementary Material

gkac1220_Supplemental_Files

## References

[1] Ghaly T.M. , Gillings M.R. Mobile DNAs as ecologically and evolutionarily independent units of life. Trends Microbiol. 2018; 26:904–912. 29885781 10.1016/j.tim.2018.05.008

[2] Koonin E.V. Viruses and mobile elements as drivers of evolutionary transitions. Philos. Trans. R. Soc. B Biol. Sci. 2016; 371:20150442. 10.1098/rstb.2015.0442 PMC4958936 27431520

[3] Kazazian H.H. Mobile elements: drivers of genome evolution. Science. 2004; 303:1626–1632. 15016989 10.1126/science.1089670

[4] San Millan A. , MacLean R.C. Fitness costs of plasmids: a limit to plasmid transmission. Microbiol. Spectr. 2017; 5:10.1128/microbiolspec.MTBP-0016-2017. PMC11687550 28944751

[5] Botelho J. , Schulenburg H. The role of integrative and conjugative elements in antibiotic resistance evolution. Trends Microbiol. 2020; 29:8–18. 32536522 10.1016/j.tim.2020.05.011

[6] Chen J. , Quiles-Puchalt N., Chiang Y.N., Bacigalupe R., Fillol-Salom A., Chee M.S.J., Fitzgerald J.R., Penadés J.R. Genome hypermobility by lateral transduction. Science. 2018; 362:207–212. 30309949 10.1126/science.aat5867

[7] Forster S.C. , Liu J., Kumar N., Gulliver E.L., Gould J.A., Escobar-Zepeda A., Mkandawire T., Pike L.J., Shao Y., Stares M.D. et al. Strain-level characterization of broad host range mobile genetic elements transferring antibiotic resistance from the human microbiome. Nat. Commun. 2022; 13:1445. 35301310 10.1038/s41467-022-29096-9 PMC8931123

[8] Dagan T. , Artzy-Randrup Y., Martin W. Modular networks and cumulative impact of lateral transfer in prokaryote genome evolution. Proc. Natl. Acad. Sci. U.S.A. 2008; 105:10039–10044. 18632554 10.1073/pnas.0800679105 PMC2474566

[9] Smillie C.S. , Smith M.B., Friedman J., Cordero O.X., David L.A., Alm E.J. Ecology drives a global network of gene exchange connecting the human microbiome. Nature. 2011; 480:241–244. 22037308 10.1038/nature10571

[10] Ellabaan M.M.H. , Munck C., Porse A., Imamovic L., Sommer M.O.A. Forecasting the dissemination of antibiotic resistance genes across bacterial genomes. Nat. Commun. 2021; 12:2435. 33893312 10.1038/s41467-021-22757-1 PMC8065159

[11] Popa O. , Hazkani-Covo E., Landan G., Martin W., Dagan T. Directed networks reveal genomic barriers and DNA repair bypasses to lateral gene transfer among prokaryotes. Genome Res. 2011; 21:599–609. 21270172 10.1101/gr.115592.110 PMC3065707

[12] Kloesges T. , Popa O., Martin W., Dagan T. Networks of gene sharing among 329 proteobacterial genomes reveal differences in lateral gene transfer frequency at different phylogenetic depths. Mol. Biol. Evol. 2011; 28:1057–1074. 21059789 10.1093/molbev/msq297 PMC3021791

[13] Acman M. , van Dorp L., Santini J.M., Balloux F. Large-scale network analysis captures biological features of bacterial plasmids. Nat. Commun. 2020; 11:2452. 32415210 10.1038/s41467-020-16282-w PMC7229196

[14] Redondo-Salvo S. , Fernández-López R., Ruiz R., Vielva L., de Toro M., Rocha E.P.C., Garcillán-Barcia M.P., de la Cruz F. Pathways for horizontal gene transfer in bacteria revealed by a global map of their plasmids. Nat. Commun. 2020; 11:3602. 32681114 10.1038/s41467-020-17278-2 PMC7367871

[15] Iranzo J. , Krupovic M., Koonin E.V. The double-stranded DNA virosphere as a modular hierarchical network of gene sharing. Mbio. 2016; 7:e00978-16. 27486193 10.1128/mBio.00978-16 PMC4981718

[16] Croucher N.J. , Coupland P.G., Stevenson A.E., Callendrello A., Bentley S.D., Hanage W.P. Diversification of bacterial genome content through distinct mechanisms over different timescales. Nat. Commun. 2014; 5:5471. 25407023 10.1038/ncomms6471 PMC4263131

[17] Khedkar S. , Smyshlyaev G., Letunic I., Maistrenko O.M., Coelho L.P., Orakov A., Forslund S.K., Hildebrand F., Luetge M., Schmidt T.S.B. et al. Landscape of mobile genetic elements and their antibiotic resistance cargo in prokaryotic genomes. Nucleic Acids Res. 2022; 50:3155–3168. 35323968 10.1093/nar/gkac163 PMC8989519

[18] Zhang Z. , Zhang Q., Wang T., Xu N., Lu T., Hong W., Penuelas J., Gillings M., Wang M., Gao W. et al. Assessment of global health risk of antibiotic resistance genes. Nat. Commun. 2022; 13:1553. 35322038 10.1038/s41467-022-29283-8 PMC8943045

[19] Partridge S.R. , Kwong S.M., Firth N., Jensen S.O. Mobile genetic elements associated with antimicrobial resistance. Clin. Microbiol. Rev. 2018; 31:e00088-17. 30068738 10.1128/CMR.00088-17 PMC6148190

[20] Wein T. , Hülter N.F., Mizrahi I., Dagan T. Emergence of plasmid stability under non-selective conditions maintains antibiotic resistance. Nat. Commun. 2019; 10:2595. 31197163 10.1038/s41467-019-10600-7 PMC6565834

[21] Rodriguez-Beltran J. , Hernandez-Beltran J.C.R., DelaFuente J., Escudero J.A., Fuentes-Hernandez A., MacLean R.C., Peña-Miller R., San Millan A. Multicopy plasmids allow bacteria to escape from fitness trade-offs during evolutionary innovation. Nat. Ecol. Evol. 2018; 2:873–881. 29632354 10.1038/s41559-018-0529-z PMC6055991

[22] Koonin E.V. , Makarova K.S., Wolf Y.I., Krupovic M. Evolutionary entanglement of mobile genetic elements and host defence systems: guns for hire. Nat. Rev. Genet. 2019; 21:119–131. 31611667 10.1038/s41576-019-0172-9

[23] Oliveira P.H. , Touchon M., Rocha E.P.C. The interplay of restriction-modification systems with mobile genetic elements and their prokaryotic hosts. Nucleic Acids Res. 2014; 42:10618–10631. 25120263 10.1093/nar/gku734 PMC4176335

[24] Roberts R.J. , Vincze T., Posfai J., Macelis D. REBASE—a database for DNA restriction and modification: enzymes, genes and genomes. Nucleic Acids Res. 2015; 43:D298–D299. 25378308 10.1093/nar/gku1046 PMC4383893

[25] Makarova K.S. , Wolf Y.I., Iranzo J., Shmakov S.A., Alkhnbashi O.S., Brouns S.J.J., Charpentier E., Cheng D., Haft D.H., Horvath P. et al. Evolutionary classification of CRISPR–Cas systems: a burst of class 2 and derived variants. Nat. Rev. Microbiol. 2020; 18:67–83. 31857715 10.1038/s41579-019-0299-x PMC8905525

[26] Millan A.S. , Peña-Miller R., Toll-Riera M., Halbert Z.V., McLean A.R., Cooper B.S., MacLean R.C. Positive selection and compensatory adaptation interact to stabilize non-transmissible plasmids. Nat. Commun. 2014; 5:5208. 25302567 10.1038/ncomms6208 PMC4208098

[27] Bondy-Denomy J. , Pawluk A., Maxwell K.L., Davidson A.R. Bacteriophage genes that inactivate the CRISPR/Cas bacterial immune system. Nature. 2013; 493:429–432. 23242138 10.1038/nature11723 PMC4931913

[28] Li Y. , Bondy-Denomy J. Anti-CRISPRs go viral: the infection biology of CRISPR-Cas inhibitors. Cell Host Microbe. 2021; 29:704–714. 33444542 10.1016/j.chom.2020.12.007 PMC8122014

[29] Mahendra C. , Christie K.A., Osuna B.A., Pinilla-Redondo R., Kleinstiver B.P., Bondy-Denomy J. Broad-spectrum anti-CRISPR proteins facilitate horizontal gene transfer. Nat. Microbiol. 2020; 5:620–629. 32218510 10.1038/s41564-020-0692-2 PMC7194981

[30] Pinilla-Redondo R. , Shehreen S., Marino N.D., Fagerlund R.D., Brown C.M., Sørensen S.J., Fineran P.C., Bondy-Denomy J. Discovery of multiple anti-CRISPRs highlights anti-defense gene clustering in mobile genetic elements. Nat. Commun. 2020; 11:5652. 33159058 10.1038/s41467-020-19415-3 PMC7648647

[31] De Oliveira D.M.P. , Forde B.M., Kidd T.J., Harris P.N.A., Schembri M.A., Beatson S.A., Paterson D.L., Walker M.J. Antimicrobial resistance in ESKAPE pathogens. Clin. Microbiol. Rev. 2020; 33:e00181-19. 32404435 10.1128/CMR.00181-19 PMC7227449

[32] Mortensen K. , Lam T.J., Ye Y. Comparison of CRISPR–Cas immune systems in healthcare-related pathogens. Front. Microbiol. 2021; 12:758782. 34759910 10.3389/fmicb.2021.758782 PMC8573248

[33] Wyres K.L. , Nguyen T.N.T., Lam M.M.C., Judd L.M., van Vinh Chau N., Dance D.A.B., Ip M., Karkey A., Ling C.L., Miliya T. et al. Genomic surveillance for hypervirulence and multi-drug resistance in invasive *Klebsiella pneumoniae* from South and Southeast Asia. Genome Med. 2020; 12:11. 31948471 10.1186/s13073-019-0706-y PMC6966826

[34] Botelho J. , Grosso F., Peixe L. Antibiotic resistance in *Pseudomonas aeruginosa* – mechanisms, epidemiology and evolution. Drug Resist. Updat. 2019; 44:100640. 31492517 10.1016/j.drup.2019.07.002

[35] Davin-Regli A. , Lavigne J.-P., Pagès J.-M. *Enterobacter* spp.: update on taxonomy, clinical aspects, and emerging antimicrobial resistance. Clin. Microbiol. Rev. 2019; 32:e00002-19. 31315895 10.1128/CMR.00002-19 PMC6750132

[36] Copin R. , Sause W.E., Fulmer Y., Balasubramanian D., Dyzenhaus S., Ahmed J.M., Kumar K., Lees J., Stachel A., Fisher J.C. et al. Sequential evolution of virulence and resistance during clonal spread of community-acquired methicillin-resistant *Staphylococcus aureus*. Proc. Natl. Acad. Sci. U.S.A. 2019; 116:1745–1754. 30635416 10.1073/pnas.1814265116 PMC6358666

[37] Gao W. , Howden B.P., Stinear T.P. Evolution of virulence in *Enterococcus faecium*, a hospital-adapted opportunistic pathogen. Curr. Opin. Microbiol. 2018; 41:76–82. 29227922 10.1016/j.mib.2017.11.030

[38] Harding C.M. , Hennon S.W., Feldman M.F. Uncovering the mechanisms of *Acinetobacter baumannii* virulence. Nat. Rev. Microbiol. 2018; 16:91–102. 29249812 10.1038/nrmicro.2017.148 PMC6571207

[39] Cassini A. , Högberg L.D., Plachouras D., Quattrocchi A., Hoxha A., Simonsen G.S., Colomb-Cotinat M., Kretzschmar M.E., Devleesschauwer B., Cecchini M. et al. Attributable deaths and disability-adjusted life-years caused by infections with antibiotic-resistant bacteria in the EU and the European Economic Area in 2015: a population-level modelling analysis. Lancet. Infect. Dis. 2018; 19:56–66. 30409683 10.1016/S1473-3099(18)30605-4 PMC6300481

[40] Botelho J. , Mourão J., Roberts A.P., Peixe L. Comprehensive genome data analysis establishes a triple whammy of carbapenemases, ices and multiple clinically relevant bacteria. Microb. Genom. 2020; 6:mgen000424. 32841111 10.1099/mgen.0.000424 PMC7660259

[41] Paauw A. , Leverstein-van Hall M.A., Verhoef J., Fluit A.C. Evolution in quantum leaps: multiple combinatorial transfers of HPI and other genetic modules in *Enterobacteriaceae*. PLoS One. 2010; 5:8662. 10.1371/journal.pone.0008662 PMC2801613 20084283

[42] Pinilla-Redondo R. , Mayo-Muñoz D., Russel J., Garrett R.A., Randau L., Sørensen S.J., Shah S.A. Type IV CRISPR-Cas systems are highly diverse and involved in competition between plasmids. Nucleic Acids Res. 2020; 48:2000–2012. 31879772 10.1093/nar/gkz1197 PMC7038947

[43] León L.M. , Park A.E., Borges A.L., Zhang J.Y., Bondy-Denomy J. Mobile element warfare via CRISPR and anti-CRISPR in *Pseudomonas aeruginosa*. Nucleic Acids Res. 2021; 49:2114–2125. 33544853 10.1093/nar/gkab006 PMC7913775

[44] Moya-Beltrán A. , Makarova K.S., Acuña L.G., Wolf Y.I., Covarrubias P.C., Shmakov S.A., Silva C., Tolstoy I., Johnson D.B., Koonin E.V. et al. Evolution of type IV CRISPR-Cas systems: insights from CRISPR loci in integrative conjugative elements of *Acidithiobacillia*. CRISPR J. 2021; 4:656–672. 34582696 10.1089/crispr.2021.0051 PMC8658065

[45] Richter M. , Rosselló-Móra R. Shifting the genomic gold standard for the prokaryotic species definition. Proc. Natl. Acad. Sci. U.S.A. 2009; 106:19126–19131. 19855009 10.1073/pnas.0906412106 PMC2776425

[46] Jain C. , Rodriguez-R L.M., Phillippy A.M., Konstantinidis K.T., Aluru S. High throughput ANI analysis of 90K prokaryotic genomes reveals clear species boundaries. Nat. Commun. 2018; 9:5114. 30504855 10.1038/s41467-018-07641-9 PMC6269478

[47] Lee M.D. GToTree: a user-friendly workflow for phylogenomics. Bioinformatics. 2019; 35:4162–4164. 30865266 10.1093/bioinformatics/btz188 PMC6792077

[48] Nguyen L.T. , Schmidt H.A., Von Haeseler A., Minh B.Q IQ-TREE: a fast and effective stochastic algorithm for estimating maximum-likelihood phylogenies. Mol. Biol. Evol. 2015; 32:268–274. 25371430 10.1093/molbev/msu300 PMC4271533

[49] Seemann T. Prokka: rapid prokaryotic genome annotation. Bioinformatics. 2014; 30:2068–2069. 24642063 10.1093/bioinformatics/btu153

[50] Gautreau G. , Bazin A., Gachet M., Planel R., Burlot L., Dubois M., Perrin A., Médigue C., Calteau A., Cruveiller S. et al. PPanGGOLiN: depicting microbial diversity via a partitioned pangenome graph. PLoS Comput. Biol. 2020; 16:e1007732. 32191703 10.1371/journal.pcbi.1007732 PMC7108747

[51] Bazin A. , Gautreau G., Médigue C., Vallenet D., Calteau A. panRGP: a pangenome-based method to predict genomic islands and explore their diversity. Bioinformatics. 2020; 36:i651–i658. 33381850 10.1093/bioinformatics/btaa792

[52] Quinlan A.R. , Hall I.M. BEDTools: a flexible suite of utilities for comparing genomic features. Bioinformatics. 2010; 26:841–842. 20110278 10.1093/bioinformatics/btq033 PMC2832824

[53] Eddy S.R. Accelerated profile HMM searches. PLoS Comput. Biol. 2011; 7:e1002195. 22039361 10.1371/journal.pcbi.1002195 PMC3197634

[54] Garcillán-Barcia M.P. , Redondo-Salvo S., Vielva L., de la Cruz F. MOBscan: automated annotation of MOB relaxases. Methods Mol. Biol. 2020; 2075:295–308. 31584171 10.1007/978-1-4939-9877-7_21

[55] Akhter S. , Aziz R.K., Edwards R.A. PhiSpy: a novel algorithm for finding prophages in bacterial genomes that combines similarity-and composition-based strategies. Nucleic Acids Res. 2012; 40:e126. 22584627 10.1093/nar/gks406 PMC3439882

[56] Steinegger M. , Söding J. Clustering huge protein sequence sets in linear time. Nat. Commun. 2018; 9:2542. 29959318 10.1038/s41467-018-04964-5 PMC6026198

[57] Zhao X. BinDash, software for fast genome distance estimation on a typical personal laptop. Bioinformatics. 2019; 35:671–673. 30052763 10.1093/bioinformatics/bty651

[58] Buchfink B. , Reuter K., Drost H.-G. Sensitive protein alignments at tree-of-life scale using DIAMOND. Nat. Methods. 2021; 18:366–368. 33828273 10.1038/s41592-021-01101-x PMC8026399

[59] Cantalapiedra C.P. , Hernández-Plaza A., Letunic I., Bork P., Huerta-Cepas J. eggNOG-mapper v2: functional annotation, orthology assignments, and domain prediction at the metagenomic scale. Mol. Biol. Evol. 2021; 38:5825–5829. 34597405 10.1093/molbev/msab293 PMC8662613

[60] Zankari E. , Hasman H., Cosentino S., Vestergaard M., Rasmussen S., Lund O., Aarestrup F.M., Larsen M.V. Identification of acquired antimicrobial resistance genes. J. Antimicrob. Chemother. 2012; 67:2640–2644. 22782487 10.1093/jac/dks261 PMC3468078

[61] Chen L. , Zheng D., Liu B., Yang J., Jin Q. VFDB 2016: hierarchical and refined dataset for big data analysis - 10 years on. Nucleic Acids Res. 2016; 44:D694–D697. 26578559 10.1093/nar/gkv1239 PMC4702877

[62] Russel J. , Pinilla-Redondo R., Mayo-Muñoz D., Shah S.A., Sørensen S.J. CRISPRCasTyper: automated identification, annotation, and classification of CRISPR-Cas Loci. CRISPR. J. 2020; 3:462–469. 33275853 10.1089/crispr.2020.0059

[63] Shmakov S.A. , Sitnik V., Makarova K.S., Wolf Y.I., Severinov K.V., Koonin E.V. The CRISPR spacer space is dominated by sequences from species-specific mobilomes. Mbio. 2017; 8:e01397-17. 28928211 10.1128/mBio.01397-17 PMC5605939

[64] Smillie C. , Garcillán-Barcia M.P., Francia M.V., Rocha E.P.C., de la Cruz F. Mobility of plasmids. Microbiol. Mol. Biol. Rev. 2010; 74:434–452. 20805406 10.1128/MMBR.00020-10 PMC2937521

[65] Nishida H. Comparative analyses of base compositions, DNA sizes, and dinucleotide frequency profiles in archaeal and bacterial chromosomes and plasmids. Int. J. Evol. Biol. 2012; 2012:342482. 22536540 10.1155/2012/342482 PMC3321278

[66] Cury J. , Touchon M., Rocha E.P.C. Integrative and conjugative elements and their hosts: composition, distribution and organization. Nucleic Acids Res. 2017; 45:8943–8956. 28911112 10.1093/nar/gkx607 PMC5587801

[67] Almpanis A. , Swain M., Gatherer D., McEwan N. Correlation between bacterial G+C content, genome size and the G+C content of associated plasmids and bacteriophages. Microb. Genom. 2018; 4:e000168. 29633935 10.1099/mgen.0.000168 PMC5989581

[68] Achtman M. , Wagner M. Microbial diversity and the genetic nature of microbial species. Nat. Rev. Microbiol. 2008; 6:431–440. 18461076 10.1038/nrmicro1872

[69] Shapiro B.J. , Friedman J., Cordero O.X., Preheim S.P., Timberlake S.C., Szabó G., Polz M.F., Alm E.J. Population genomics of early events in the ecological differentiation of bacteria. Science. 2012; 336:48–51. 22491847 10.1126/science.1218198 PMC3337212

[70] Galperin M.Y. , Wolf Y.I., Makarova K.S., Alvarez R.V., Landsman D., Koonin E.V. COG database update: focus on microbial diversity, model organisms, and widespread pathogens. Nucleic Acids Res. 2021; 49:D274–D281. 33167031 10.1093/nar/gkaa1018 PMC7778934

[71] Galperin M.Y. , Kristensen D.M., Makarova K.S., Wolf Y.I., Koonin E.V. Microbial genome analysis: the COG approach. Brief. Bioinform. 2019; 20:1063–1070. 28968633 10.1093/bib/bbx117 PMC6781585

[72] Enault F. , Briet A., Bouteille L., Roux S., Sullivan M.B., Petit M.-A. Phages rarely encode antibiotic resistance genes: a cautionary tale for virome analyses. ISME J. 2017; 11:237–247. 27326545 10.1038/ismej.2016.90 PMC5315482

[73] John J. , George S., Nori S.R.C., Nelson-Sathi S. Phylogenomic analysis reveals the evolutionary route of resistant genes in *Staphylococcus aureus*. Genome Biol. Evol. 2019; 11:2917–2926. 31589296 10.1093/gbe/evz213 PMC6808081

[74] Lam M.M.C. , Wick R.R., Wyres K.L., Gorrie C.L., Judd L.M., Jenney A.W.J., Brisse S., Holt K.E. Genetic diversity, mobilisation and spread of the yersiniabactin-encoding mobile element ICEKp in *Klebsiella pneumoniae* populations. Microb. Genom. 2018; 4:e000196. 29985125 10.1099/mgen.0.000196 PMC6202445

[75] Pinilla-Redondo R. , Russel J., Mayo-Muñoz D., Shah S.A., Garrett R.A., Nesme J., Madsen J.S., Fineran P.C., Sørensen S.J. CRISPR-Cas systems are widespread accessory elements across bacterial and archaeal plasmids. Nucleic Acids Res. 2021; 50:4315–4328. 10.1093/nar/gkab859 PMC9071438 34606604

[76] van Belkum A. , Soriaga L.B., LaFave M.C., Akella S., Veyrieras J.-B., Barbu E.M., Shortridge D., Blanc B., Hannum G., Zambardi G. et al. Phylogenetic distribution of CRISPR-Cas systems in antibiotic-resistant *Pseudomonas aeruginosa*. Mbio. 2015; 6:e01796-15. 26604259 10.1128/mBio.01796-15 PMC4669384

[77] Wheatley R.M. , MacLean R.C. CRISPR-Cas systems restrict horizontal gene transfer in *Pseudomonas aeruginosa*. ISME J. 2020; 15:1420–1433. 33349652 10.1038/s41396-020-00860-3 PMC8105352

[78] Pursey E. , Dimitriu T., Paganelli F.L., Westra E.R., Houte S.van CRISPR-Cas is associated with fewer antibiotic resistance genes in bacterial pathogens. Philos. Trans. R. Soc. B. 2022; 377:20200464. 10.1098/rstb.2020.0464 PMC8628084 34839714

[79] Halary S. , Leigh J.W., Cheaib B., Lopez P., Bapteste E. Network analyses structure genetic diversity in independent genetic worlds. Proc. Natl. Acad. Sci. U.S.A. 2010; 107:127–132. 20007769 10.1073/pnas.0908978107 PMC2806761

[80] Cury J. , Oliveira P.H., de la Cruz F., Rocha E.P.C. Host range and genetic plasticity explain the coexistence of integrative and extrachromosomal mobile genetic elements. Mol. Biol. Evol. 2018; 35:2230–2239. 29905872 10.1093/molbev/msy123 PMC6107060

[81] Rollie C. , Chevallereau A., Watson B.N.J., Chyou T., Fradet O., McLeod I., Fineran P.C., Brown C.M., Gandon S., Westra E.R. Targeting of temperate phages drives loss of type I CRISPR–Cas systems. Nature. 2020; 578:149–153. 31969710 10.1038/s41586-020-1936-2 PMC7007301

[82] Al-Shayeb B. , Sachdeva R., Chen L.-X., Ward F., Munk P., Devoto A., Castelle C.J., Olm M.R., Bouma-Gregson K., Amano Y. et al. Clades of huge phages from across Earth's ecosystems. Nature. 2020; 578:425–431. 32051592 10.1038/s41586-020-2007-4 PMC7162821

[83] Liu M. , Li X., Xie Y., Bi D., Sun J., Li J., Tai C., Deng Z., Ou H.-Y. ICEberg 2.0: an updated database of bacterial integrative and conjugative elements. Nucleic Acids Res. 2018; 47:D660–D665. 10.1093/nar/gky1123 PMC6323972 30407568

[84] Doron S. , Melamed S., Ofir G., Leavitt A., Lopatina A., Keren M., Amitai G., Sorek R. Systematic discovery of antiphage defense systems in the microbial pangenome. Science. 2018; 359:eaar4120. 29371424 10.1126/science.aar4120 PMC6387622

[85] Fillol-Salom A. , Martínez-Rubio R., Abdulrahman R.F., Chen J., Davies R., Penadés J.R. Phage-inducible chromosomal islands are ubiquitous within the bacterial universe. ISME J. 2018; 12:2114–2128. 29875435 10.1038/s41396-018-0156-3 PMC6092414

