# Supplementary material for: The ESKAPE mobilome contributes to the spread of antimicrobial resistance and CRISPR-mediated conflict between mobile genetic elements
Source: Nucleic Acids Res. 2023 Jan 5;51(1):236–52. doi: 10.1093/nar/gkac1220 (PMC9841420; doi:10.1093/nar/gkac1220)
Supplement: gkac1220_Supplemental_Files [file gkac1220_supplemental_files.zip › Supl_tables_legends.pdf]

Supplementary Table S1. Average nucleotide identity based on MUMmer (ANIm) matrices showing all vs. all comparisons between genomes used in this study. Each ESKAPE species is represented in a separate sheet.

Supplementary Table S2. General characteristics for the 1746 ESKAPE genomes used in this study.

Supplementary Table S3. Annotations for the ESKAPE MGEs proteome and widespread proteins.

Supplementary Table S4. List of antimicrobial resistance genes found across the ESKAPE mobilome.

Supplementary Table S5. List of virulence genes found across the ESKAPE mobilome.

Supplementary Table S6. CRISPR-Cas systems found across the MGEs and masked (MGE-free) genomes from the ESKAPE pathogens.

Supplementary Table S7. Total number of genes and presence/absence of CRISPR-Cas systems across plasmids and ICEs/IMEs across the ESKAPE pathogens.

Supplementary Table S8. Anti-CRISPRs found across the MGEs and masked (MGE-free) genomes from the ESKAPE pathogens.

Supplementary Table S9. MGE spacers and corresponding targets across the ESKAPE mobilome. Only matches equal or above 95% identity and coverage were considered for this analysis.

Supplementary Table S10. List of the ESKAPE mobilome genes and corresponding proteins that are targeted by CRISPR spacers found across the MGEs.

Supplementary Table S11. Masked genome (MGE-free) spacers and corresponding targets across the ESKAPE mobilome. Only matches equal or above 95% identity and coverage were considered for this analysis.

Supplementary Table S12. CRISPR-Cas systems found across the excluded *E. faecium* genomes, due to average nucleotide identity (ANI) values below the 95% cutoff for species delineation.
