# Supplementary material for: The ESKAPE mobilome contributes to the spread of antimicrobial resistance and CRISPR-mediated conflict between mobile genetic elements
Source: Nucleic Acids Res. 2023 Jan 5;51(1):236–52. doi: 10.1093/nar/gkac1220 (PMC9841420; doi:10.1093/nar/gkac1220)
Supplement: gkac1220_Supplemental_Files [file gkac1220_supplemental_files.zip › SUPPLEMENTARY FIGURES.pdf]

SUPPLEMENTARY FIGURES

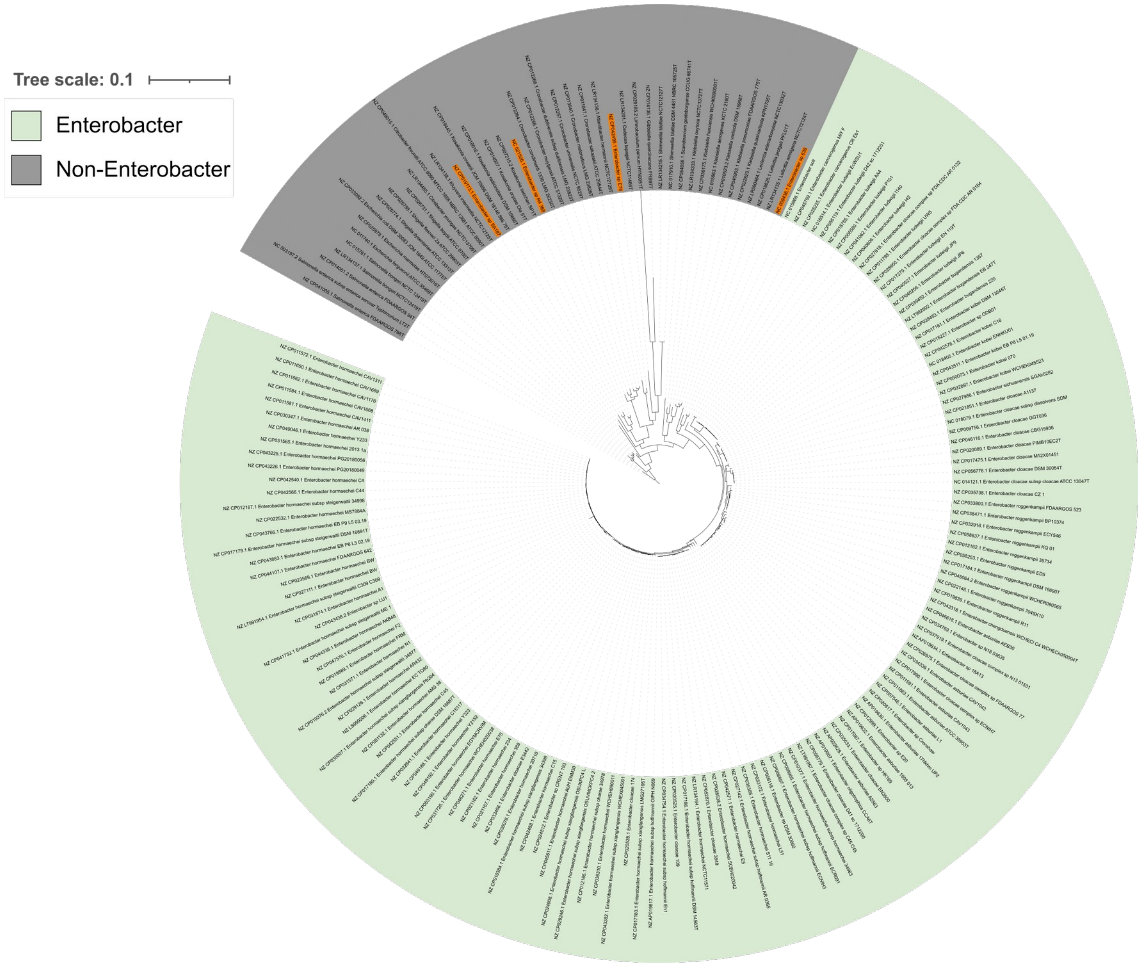

Figure S1. Maximum likelihood tree representing type strains belonging to the Enterobacteriaceae family and all complete genomes classified as Enterobacter. Genomes that are positioned in the non-Enterobacter cluster (gray), and poorly classified as belonging to the Enterobacter genus, were highlighted in orange.

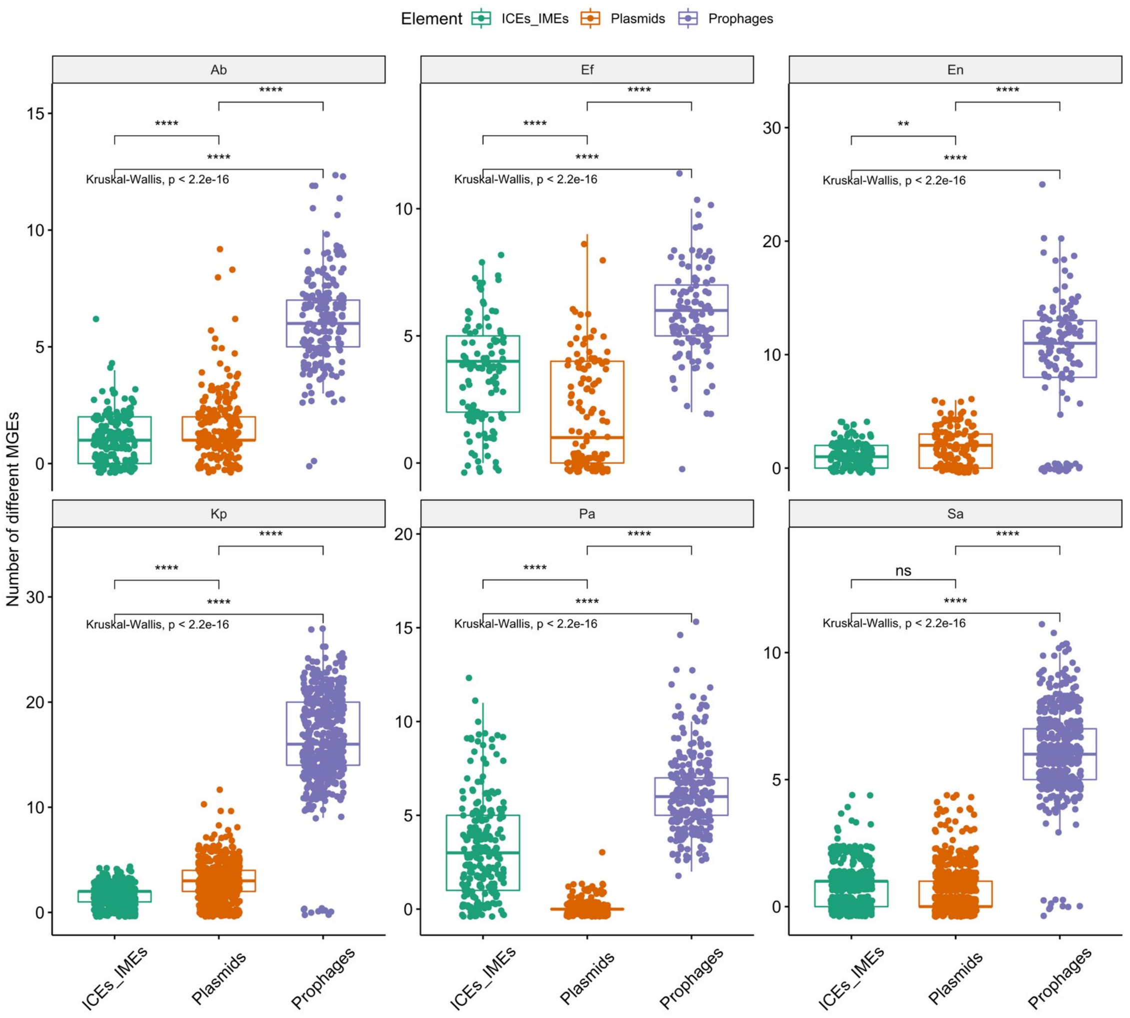

Figure S2. Boxplots representing the number of prophages, ICEs/IMEs, and plasmids per genome across the ESKAPE pathogens. Ab, *A. baumannii*; Ef, *E. faecium*; En, *Enterobacter* sp.; Kp, *K. pneumoniae*; Pa, *P. aeruginosa*; Sa, *S. aureus*. Values above 0.05 were considered as non-significant (ns). We used the following convention for symbols indicating statistical significance: * for p <= 0.05, ** for p <= 0.01, *** for p <= 0.001, and **** for p <= 0.0001.

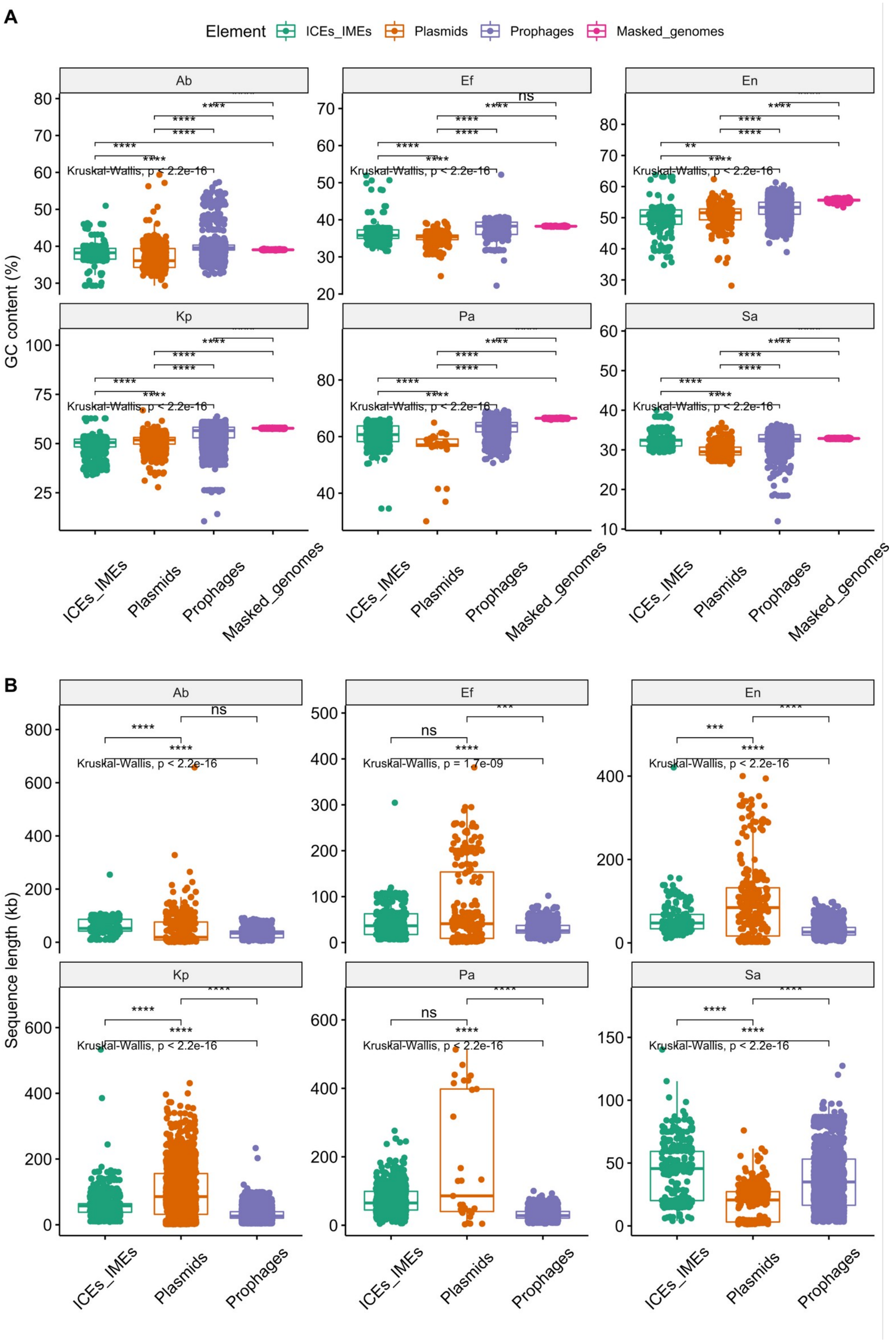

Figure S3. Variation in **A)** GC content and **B)** sequence length between MGEs and masked genomes. Ab, *A. baumannii*; Ef, *E. faecium*; En, *Enterobacter* sp.; Kp, *K. pneumoniae*; Pa, *P. aeruginosa*; Sa, *S. aureus*. Values above 0.05 were considered as non-significant (ns). We used the following convention for symbols indicating statistical significance: * for p <= 0.05, ** for p <= 0.01, *** for p <= 0.001, and **** for p <= 0.0001.

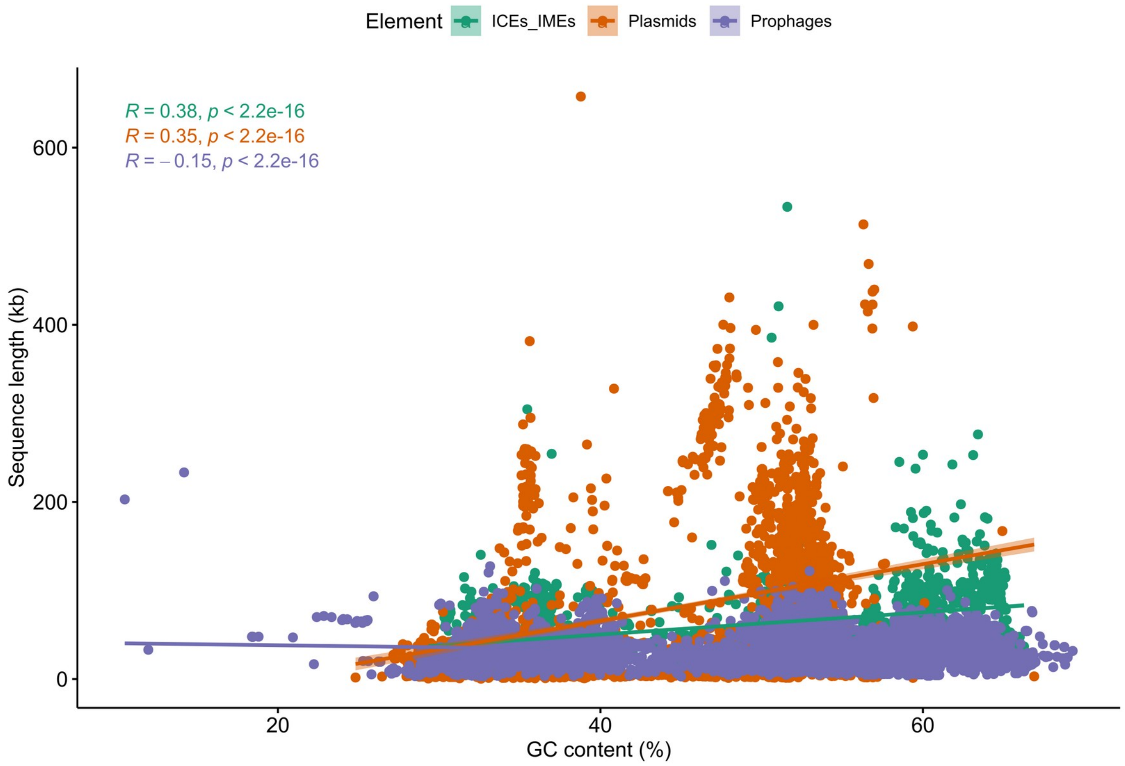

Figure S4. Scatter plot between the different MGEs' sequence length and GC content. Regression lines for each MGE type are shown in different colours. Confidence interval is displayed around smooth. Level of confidence interval is 0.95.

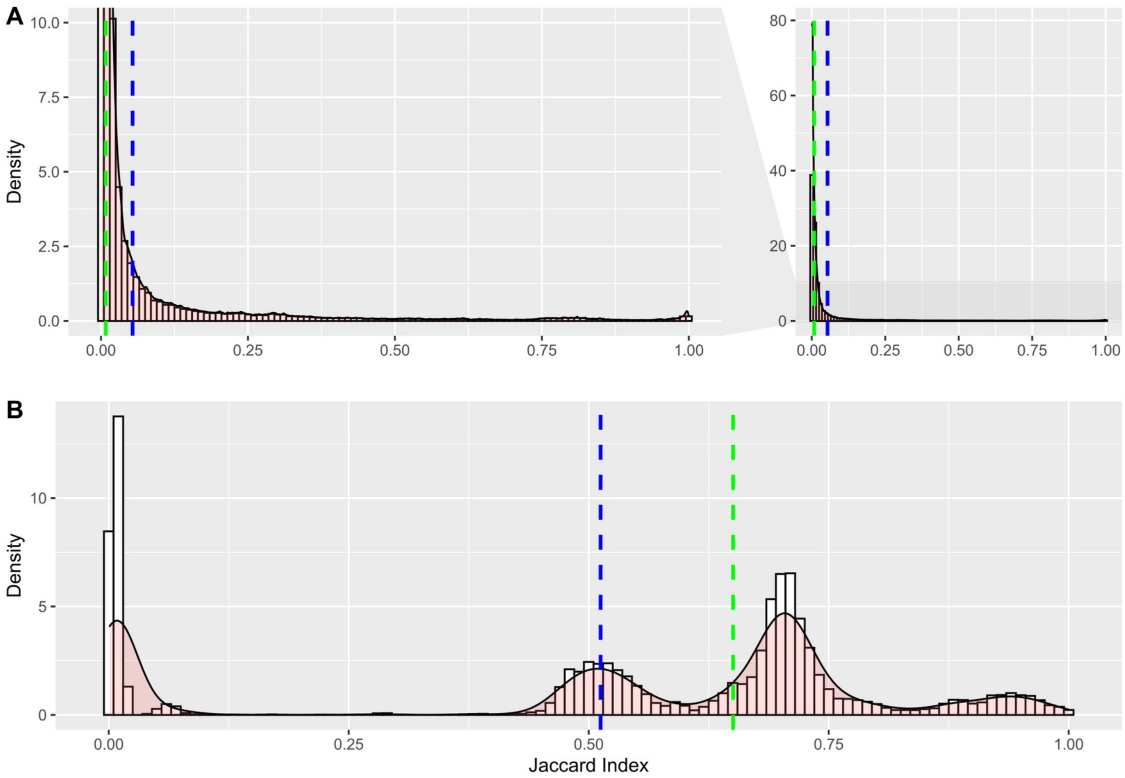

Figure S5. Density plot showing the distribution of Jaccard index (JI) values for the **A)** MGE and **B)** masked genomes network. The mean distribution for the JI is shown in blue, while the median is shown in green. In **A)**, density values from 0-10 were zoomed in and shown in the left.

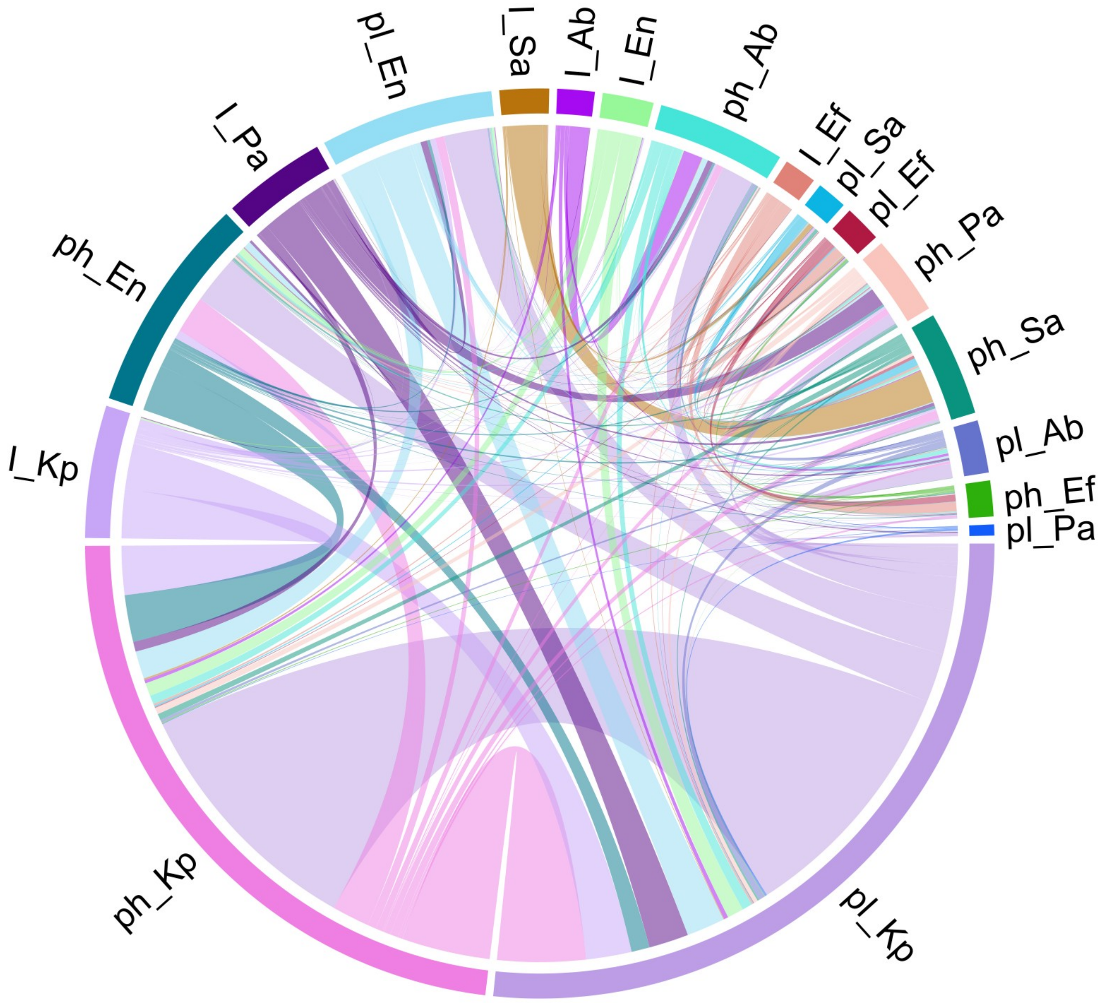

Figure S6. Chord diagram showing DNA sharing events between different ESKAPE pathogens. The width of sectors represents total number of inferred interactions between two different MGE/ESKAPE pairs. The width of links are proportional to the number of inferred DNA sharing events. Interactions between the same ESKAPE/MGE pairs were excluded. Ab, *A. baumannii*; Ef, *E. faecium*; En, *Enterobacter* sp.; Kp, *K. pneumoniae*; Pa, *P. aeruginosa*; Sa, *S. aureus*.

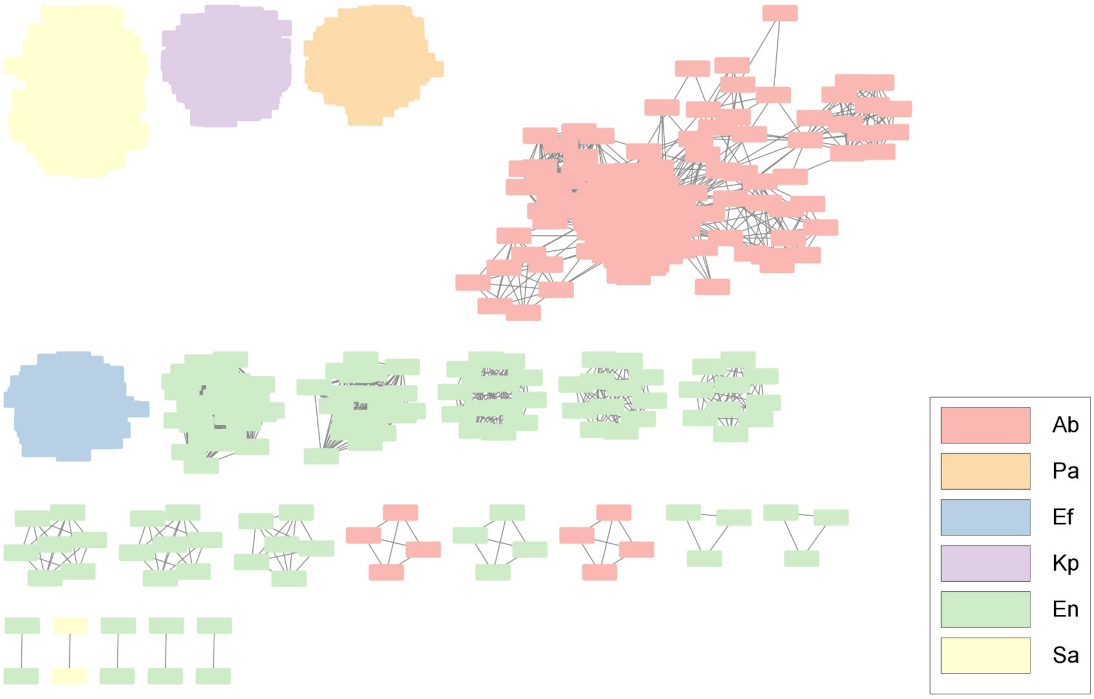

Figure S7. Network of clustered masked genomes grouped by ESKAPE pathogen, using the mean Jaccard index as a threshold. Each masked genome is represented by a node, connected by edges according to the pairwise distances between all masked genome pairs. Ab, *A. baumannii*; Ef, *E. faecium*; En, *Enterobacter* sp.; Kp, *K. pneumoniae*; Pa, *P. aeruginosa*; Sa, *S. aureus*.

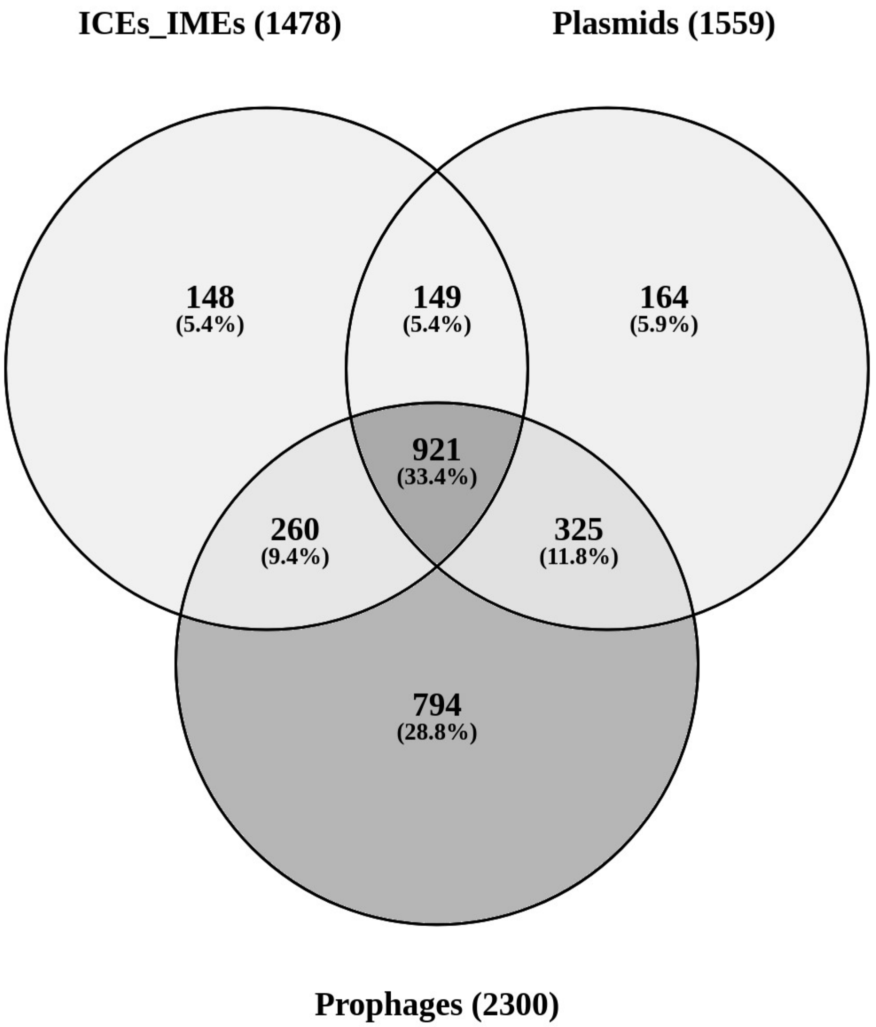

Figure S8. COGs identified in ESKAPE MGEs. Venn diagram illustrating the distribution of 2761 different COGs identified in ESKAPE ICEs/IMEs, plasmids and phages. The number of COGs detected for each MGE type is indicated in parenthesis. Note that the numbers displayed in the figure do not correspond to the total number of proteins with an identifiable COG in the MGE sequences analysed but to the distinct COG definitions (e.g. COG0105) detected in the MGE proteomes.

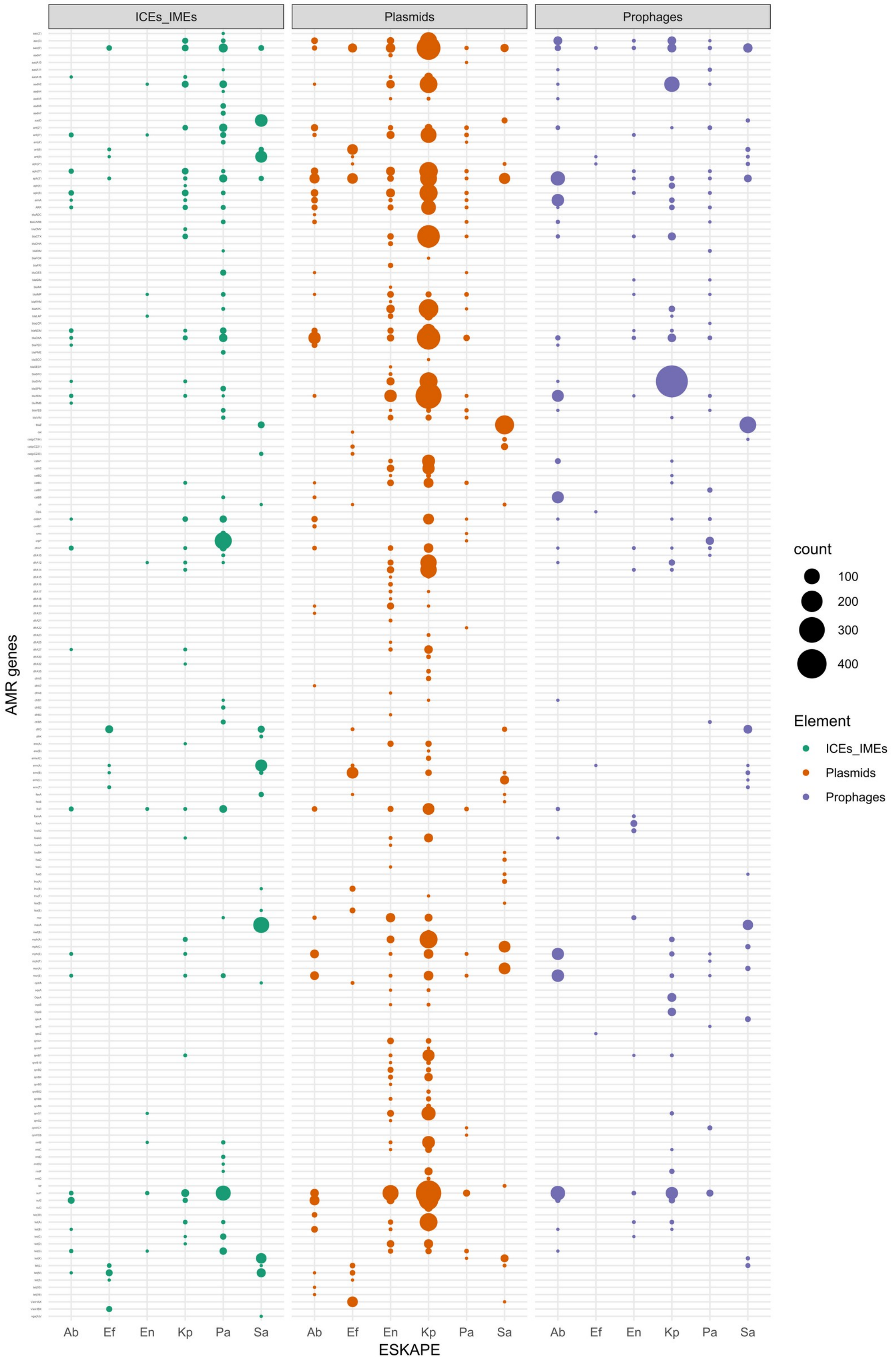

Figure S9. Distribution of antimicrobial resistance (AMR) genes across different ESKAPE pathogens and mobile elements. The size of the circles is proportional to absolute counts. Ab, *A. baumannii*; Ef, *E. faecium*; En, *Enterobacter* sp.; Kp, *K. pneumoniae*; Pa, *P. aeruginosa*; Sa, *S. aureus*.

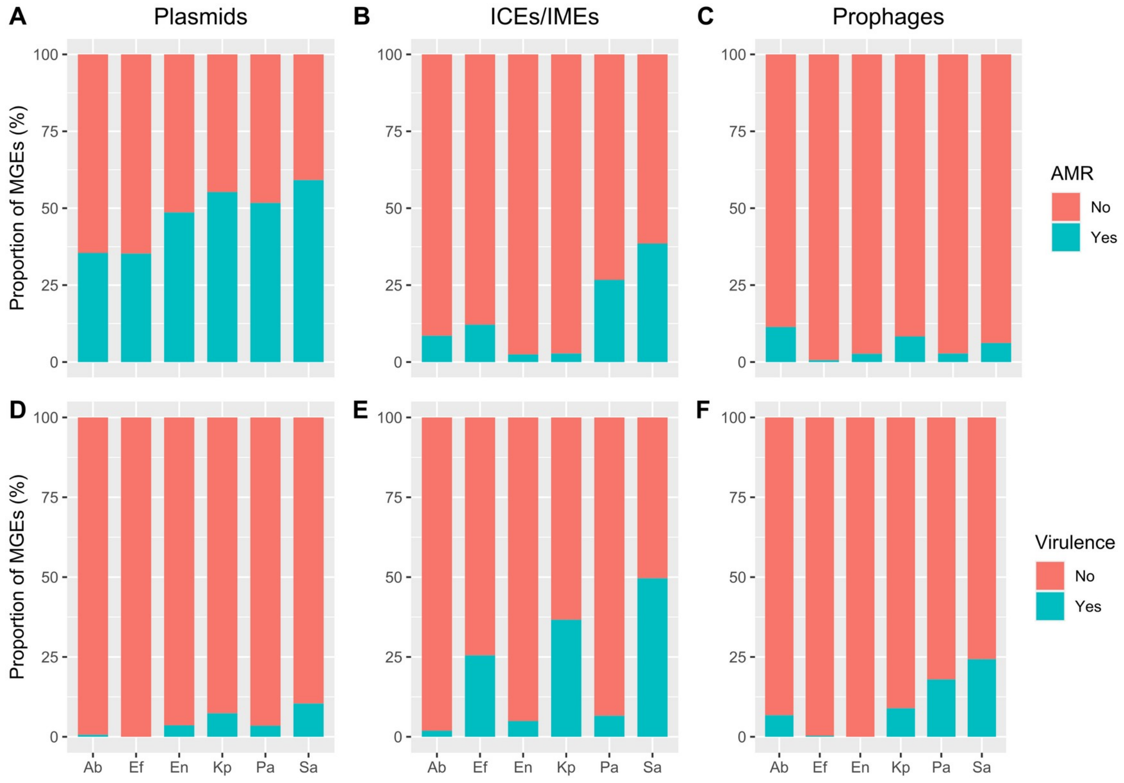

Figure S10. Proportion of mobile genetic elements (MGEs) carrying antimicrobial resistance (AMR) genes, identified in **A)** plasmids, **B)** ICEs/IMEs, and **C)** prophages, and virulence genes identified in **D)** plasmids, **E)** ICEs/IMEs, and **F)** prophages. A separate proportion is shown for the different ESKAPE pathogens. Each MGE was considered as positive for AMR or virulence genes if at least one of these genes were identified. Ab, *A. baumannii*; Ef, *E. faecium*; En, *Enterobacter* sp.; Kp, *K. pneumoniae*; Pa, *P. aeruginosa*; Sa, *S. aureus*.

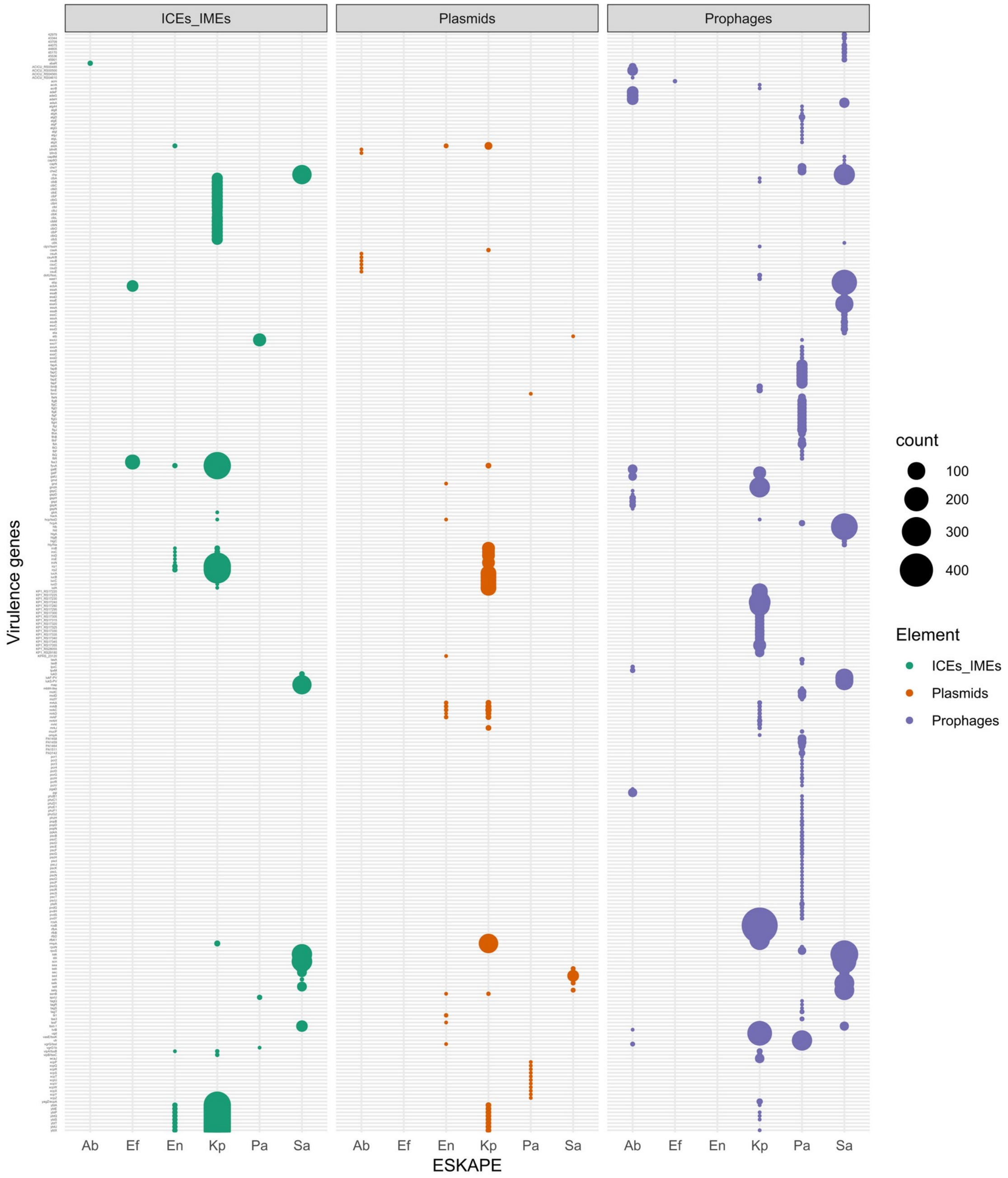

Figure S11. Distribution of virulence genes across different ESKAPE pathogens and mobile elements. The size of the circles is proportional to absolute counts. Ab, *A. baumannii*; Ef, *E. faecium*; En, *Enterobacter* sp.; Kp, *K. pneumoniae*; Pa, *P. aeruginosa*; Sa, *S. aureus*.

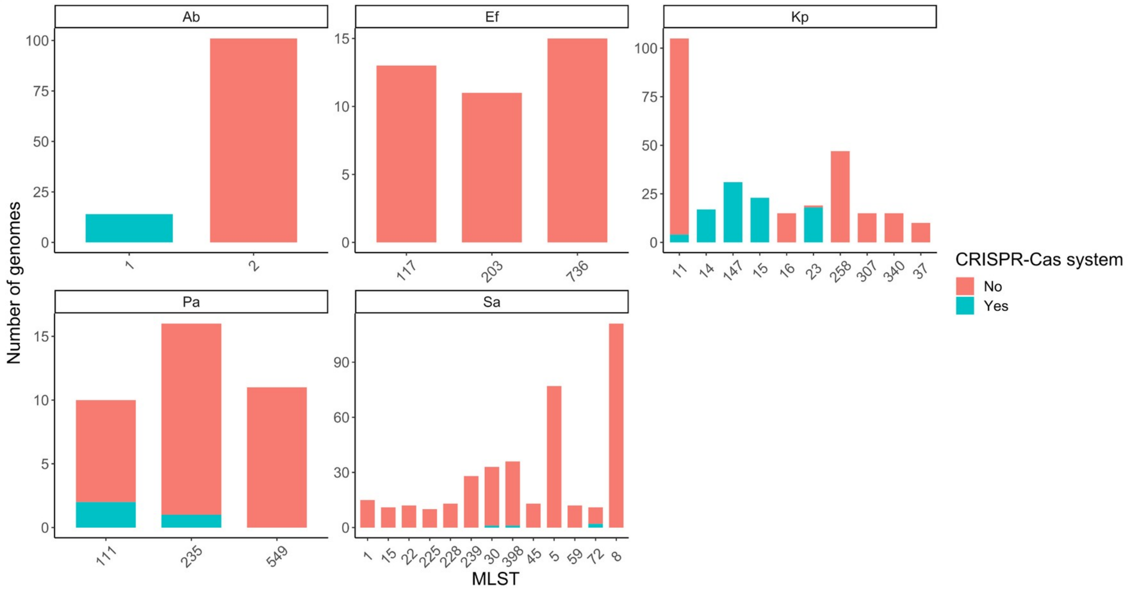

Figure S12. Number of ESKAPE genomes split by MLST profiles in the x-axis. For visualization purposes, only MLST profiles with at least 10 genomes in each ESKAPE pathogen are shown. Bar plots are coloured according to the presence or absence of CRISPR-Cas systems. Ab, *A. baumannii*; Ef, *E. faecium*; En, *Enterobacter* sp.; Kp, *K. pneumoniae*; Pa, *P. aeruginosa*; Sa, *S. aureus*.

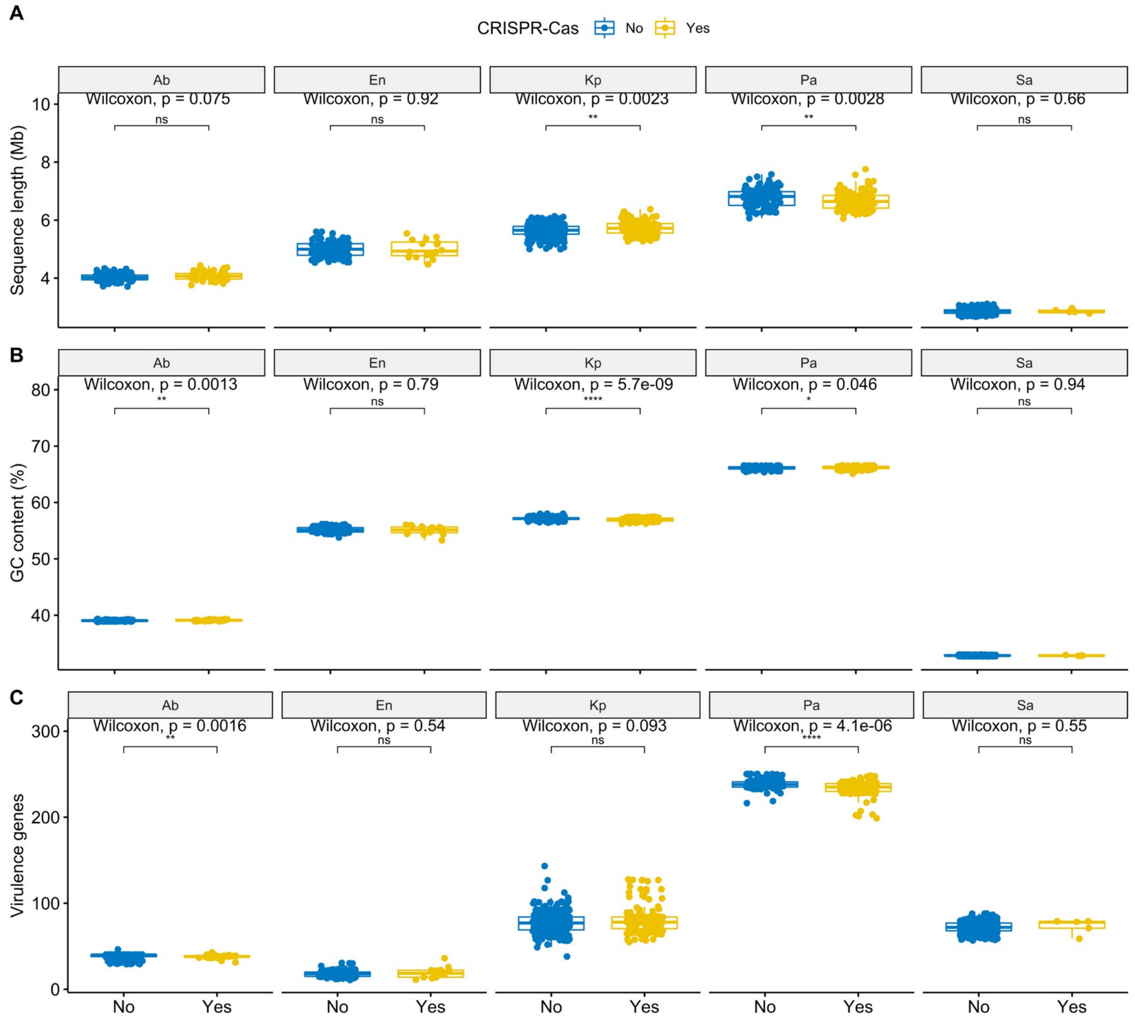

Figure S13. Boxplots comparing the **A)** sequence length, **B)** GC content, and **C)** the distribution of virulence genes in pairs of conspecific ESKAPE pathogens, with and without CRISPR-Cas systems. Values above 0.05 were considered as non-significant (ns). We used the following convention for symbols indicating statistical significance: * for p <= 0.05, ** for p <= 0.01, *** for p <= 0.001, and **** for p <= 0.0001. Ab, *A. baumannii*; En, *Enterobacter* sp.; Kp, *K. pneumoniae*; Pa, *P. aeruginosa*; Sa, *S. aureus*.

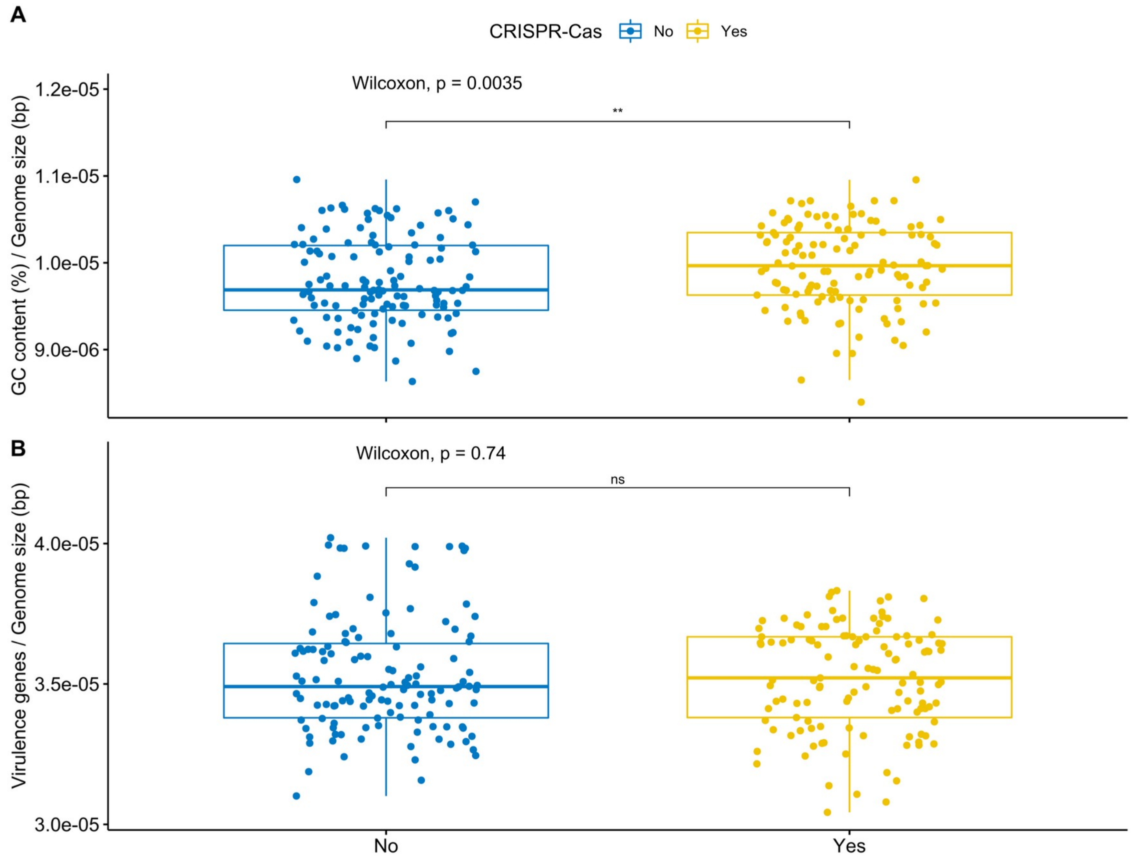

Figure S14. Boxplots comparing CRISPR-Cas positive and negative *P. aeruginosa* genomes, with **A)** GC content normalized to sequence length of each genome and **B)** virulence genes normalized to sequence length of each genome. Values above 0.05 were considered as non-significant (ns). We used the following convention for symbols indicating statistical significance: * for p <= 0.05, ** for p <= 0.01, *** for p <= 0.001, and **** for p <= 0.0001.

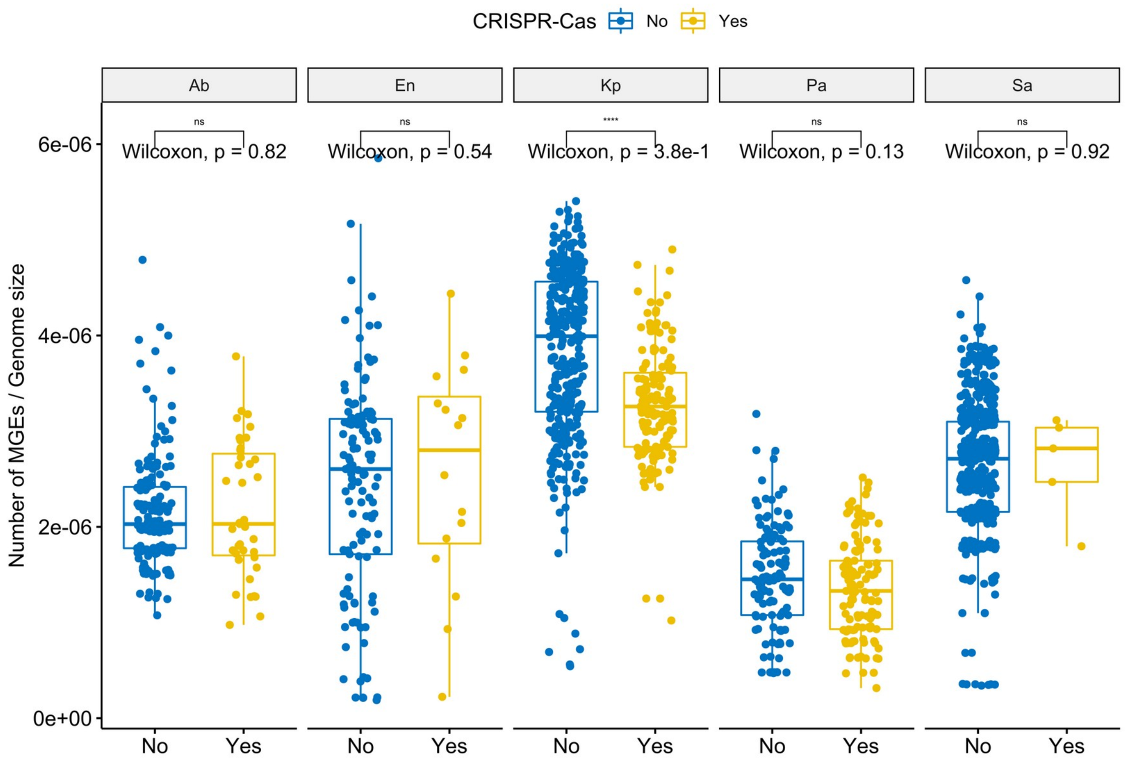

Figure S15. Boxplots compare the number of MGEs per genome size present in pairs of conspecific ESKAPE pathogens, with and without CRISPR-Cas systems. Values above 0.05 were considered as non-significant (ns). We used the following convention for symbols indicating statistical significance: * for p <= 0.05, ** for p <= 0.01, *** for p <= 0.001, and **** for p <= 0.0001. Ab, *A. baumannii*; En, *Enterobacter* sp.; Kp, *K. pneumoniae*; Pa, *P. aeruginosa*; Sa, *S. aureus*.

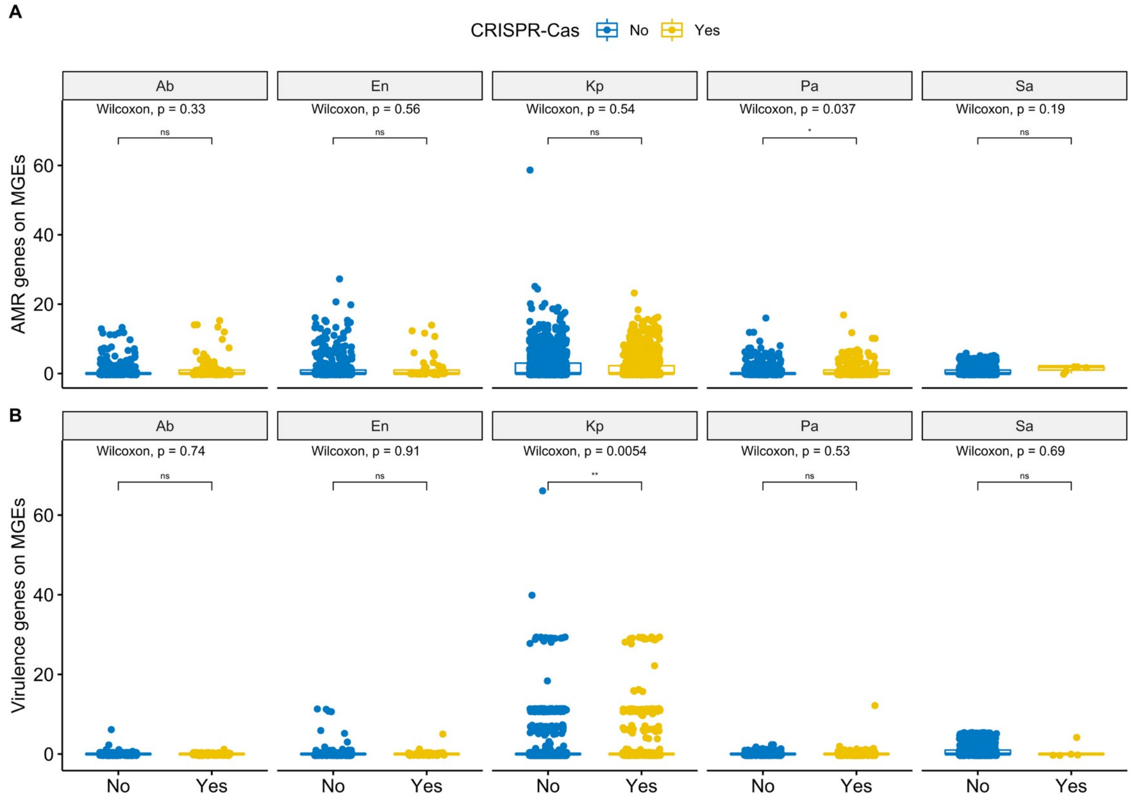

Figure S16. Boxplots comparing pairs of conspecific ESKAPE pathogens, with and without CRISPR-Cas systems, regarding plasmid- and ICE/IME-encoded **A**) antimicrobial resistance (AMR) and **B**) virulence genes. Values above 0.05 were considered as non-significant (ns). We used the following convention for symbols indicating statistical significance: * for p <= 0.05, ** for p <= 0.01, *** for p <= 0.001, and **** for p <= 0.0001. Ab, *A. baumannii*; En, *Enterobacter* sp.; Kp, *K. pneumoniae*; Pa, *P. aeruginosa*; Sa, *S. aureus*.

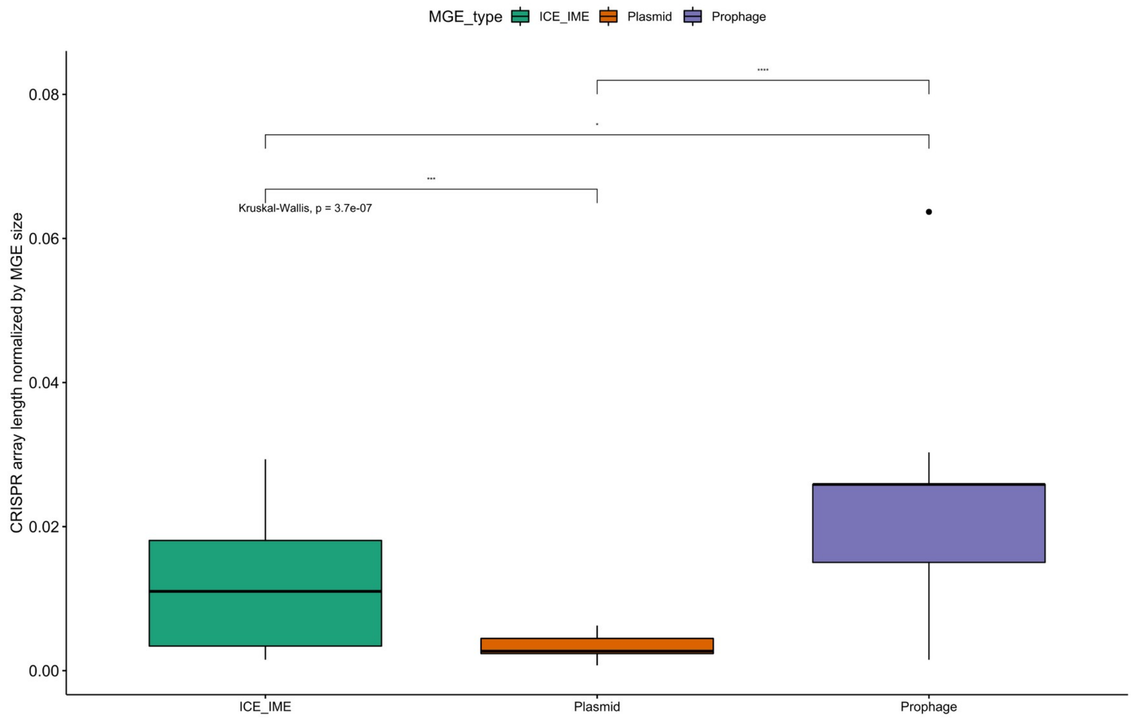

Figure S17. Boxplots comparing the CRISPR array length normalized by MGE size across all ESKAPE pathogens. We used the following convention for symbols indicating statistical significance: * for p <= 0.05, ** for p <= 0.01, *** for p <= 0.001, and **** for p <= 0.0001.

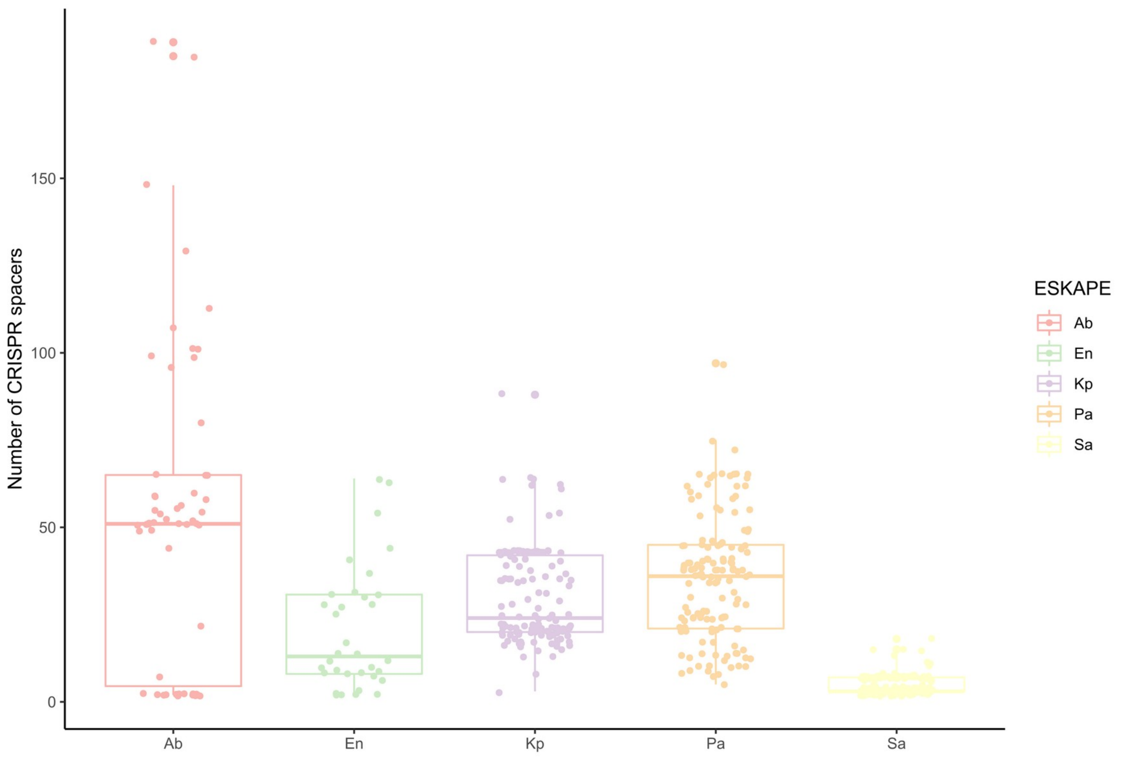

Figure S18. Absolute number of CRISPR spacers per masked genome identified across the ESKAPE pathogens.
